# Supplementary material for: What is the coverage of retina screening services for people with diabetes? Protocol for a systematic review and meta-analysis
Source: BMJ Open. 2024 Jan 30;14(1):e081123. doi: 10.1136/bmjopen-2023-081123 (PMC10828834; doi:10.1136/bmjopen-2023-081123)
Supplement: Supplementary data [file bmjopen-2023-081123supp002.pdf]

Annex 2: Search Strategy

Database: MEDLINE

1. exp Diabetes Mellitus/
2. exp Diabetes Complications/
3. Diabetic Retinopathy/
4. ((diabet$ or proliferative or non-proliferative) adj4 retinopath$).tw.
5. diabetic retinopathy.kw.
6. (diabet$ adj3 (eye$ or vision or visual$ or sight$)).tw.
7. (retinopath$ adj3 (eye$ or vision or visual$ or sight$)).tw.
8. (DR adj3 (eye$ or vision or visual$ or sight$)).tw.
9. (diabet$ adj3 macula$ adj3 oedema).tw.
10. (diabet$ adj3 macula$ adj3 edema).tw.
11. (DMO or DME).tw.
12. or/1-11
13. Vision Screening/
14. 12 and 13
15. Mass Screening/
16. or/3-11
17. 15 and 16
18. 14 or 17
19. ((eye$ or retina or retinal) adj3 (screen$ or exam$ or check$)).tw.
20. (diabet$ adj2 eye adj2 care).tw.
21. (diabet$ adj2 eye adj2 service$).tw.
22. or/19-21
23. 12 and 22
24. 18 or 23
25. (pregnan$ or gestational).ti.
26. artificial intelligence/
27. deep learning/
28. exp machine learning/
29. "neural networks (computer)"/
30. fuzzy logic/
31. (artificial adj1 intelligence).ti.
32. ((deep or machine or transfer) adj2 learning).ti.
33. ((deep or convolutional or neural) adj3 network$).ti.
34. (retinopath$ adj2 prematurity).tw.
35. (macular adj2 degeneration).ti.
36. (laser or photocoagulation or intravitreal or VEGF or bevacizumab or injection or dexamethasone or triamcinolone).ti.
37. (neuropathy or nephropathy or vascular or microvascular).ti.
38. (animal or primate or rat or rats or mouse or mice or pig).ti.
39. or/25-38
40. 24 not 39
41. case reports/
42. (case adj3 report$).tw.
43. or/41-42
44. 40 not 43
45. limit 44 to yr="2000 -Current"

Database: Embase

1. exp diabetes mellitus/
2. diabetic retinopathy/
3. ((diabet$ or proliferative or non-proliferative) adj4 retinopath$).tw.
4. diabetic retinopathy.kw.
5. (diabet$ adj3 (eye$ or vision or visual$ or sight$)).tw.
6. (retinopath$ adj3 (eye$ or vision or visual$ or sight$)).tw.
7. (DR adj3 (eye$ or vision or visual$ or sight$)).tw.
8. (diabet$ adj3 macula$ adj3 oedema).tw.
9. (diabet$ adj3 macula$ adj3 edema).tw.
10. (DMO or DME).tw.
11. or/1-10
12. exp vision test/
13. 11 and 12
14. mass screening/
15. or/2-10
16. 14 and 15
17. 13 or 16
18. ((eye$ or retina or retinal) adj3 (screen$ or exam$ or check$)).tw.
19. (diabet$ adj2 eye adj2 care).tw.
20. (diabet$ adj2 eye adj2 service$).tw.
21. or/18-20
22. 11 and 21
23. 17 or 22
24. (pregnan$ or gestational).ti.
25. artificial intelligence/
26. deep learning/
27. exp machine learning/
28. "neural networks (computer)"/
29. fuzzy logic/
30. (artificial adj1 intelligence).ti.
31. ((deep or machine or transfer) adj2 learning).ti.
32. ((deep or convolutional or neural) adj3 network$).ti.
33. (retinopath$ adj2 prematurity).tw.
34. (macular adj2 degeneration).ti.
35. (laser or photocoagulation or intravitreal or VEGF or bevacizumab or injection or dexamethasone).ti.
36. (neuropathy or nephropathy or vascular or microvascular).ti.
37. (animal or primate or rat or rats or mouse or mice or pig).ti.
38. or/24-37
39. 23 not 38
40. exp case report/
41. (case adj3 report$).tw.
42. or/40-41
43. 39 not 42
44. limit 43 to conference abstract status
45. 43 not 44
46. limit 45 to yr="2000 -Current"

Database: Global Health

1. Screening/
2. Diabetic Retinopathy/
3. ((diabet$ or proliferative or non-proliferative) adj4 retinopath$).tw.
4. (diabet$ adj3 (eye$ or vision or visual$ or sight$)).tw.
5. (retinopath$ adj3 (eye$ or vision or visual$ or sight$)).tw.
6. (DR adj3 (eye$ or vision or visual$ or sight$)).tw.
7. (diabet$ adj3 macula$ adj3 oedema).tw.
8. (diabet$ adj3 macula$ adj3 edema).tw.
9. (DMO or DME).tw.
10. or/2-9
11. 1 and 10
12. Diabetes Mellitus/
13. ((eye$ or retina or retinal) adj3 (screen$ or exam$ or check$)).tw.
14. (diabet$ adj2 eye adj2 care).tw.
15. (diabet$ adj2 eye adj2 service$).tw.
16. or/13-15
17. 10 or 12
18. 16 and 17
19. 11 or 18
20. (pregnan$ or gestational).ti.
21. artificial intelligence/
22. exp machine learning/
23. neural networks/
24. fuzzy logic/
25. (artificial adj1 intelligence).ti.
26. ((deep or machine or transfer) adj2 learning).ti.
27. ((deep or convolutional or neural) adj3 network$).ti.
28. (retinopath$ adj2 prematurity).tw.
29. (macular adj2 degeneration).ti.
30. (laser or photocoagulation or intravitreal or VEGF or bevacizumab or injection or dexamethasone).ti.
31. (neuropathy or nephropathy or vascular or microvascular).ti.
32. (animal or primate or rat or rats or mouse or mice or pig).ti.
33. or/20-32
34. 19 not 33
35. case reports/
36. (case adj3 report$).tw.
37. or/35-36
38. 34 not 37
39. limit 38 to yr="2000 -Current"

Database: CENTRAL on Cochrane Library

1. #1 MeSH descriptor: [Diabetes Mellitus] explode all trees
2. #2 MeSH descriptor: [Diabetes Complications] explode all trees
3. #3 MeSH descriptor: [Diabetic Retinopathy] this term only
4. #4 (diabet* or proliferative or non-proliferative) NEAR/4 retinopath*
5. #5 diabet* NEAR/3 (eye* or vision or visual* or sight*)
6. #6 retinopath* NEAR/3 (eye* or vision or visual* or sight*)
7. #7 DR NEAR/3 (eye* or vision or visual* or sight*)
8. #8 (diabet* NEAR/3 macula* NEAR/3 oedema)
9. #9 (diabet* NEAR/3 macula* NEAR/3 edema)
10. #10 DMO or DME
11. #11 #1 or #2 or #3 or #4 or #5 or #6 or #7 or #8 or #9 or #10
12. #12 MeSH descriptor: [Vision Screening] this term only
13. #13 #11 and #12
14. #14 MeSH descriptor: [Mass Screening] this term only
15. #15 #3 or #4 or #5 or #6 or #7 or #8 or #9 or #10
16. #16 #14 and #15
17. #17 #13 or #16
18. #18 (eye* or retina or retinal) NEAR/3 (screen* or exam* or check*)
19. #19 (diabet* NEAR/2 eye NEAR/2 care)
20. #20 (diabet* NEAR/2 eye NEAR/2 service*)
21. #21 #18 or #19 or #20
22. #22 #17 and #21 with Publication Year from 2000 to 2023, in Trials

Database: Web of Science - Science Citation Index Expanded (SCI-EXPANDED), Social Sciences Citation Index (SSCI), Emerging Sources Citation Index (ESCI)

1. 1: Screen* (Topic)
2. 2: TS=((diabet* or proliferative or non-proliferative) NEAR/4 retinopath*)
3. 3: TS= (diabet* NEAR/3 (eye* or vision or visual* or sight*))
4. 4: TS= (retinopath* NEAR/3 (eye* or vision or visual* or sight*))
5. 5: TS= (DR NEAR/3 (eye* or vision or visual* or sight*))
6. 6: TS= (diabet* NEAR/3 macula* NEAR/3 oedema)
7. 7: TS= (diabet* NEAR/3 macula* NEAR/3 edema)
8. 8: TS= (DMO or DME)
9. 9: #8 OR #7 OR #6 OR #5 OR #4 OR #3 OR #2
10. 10: #9 AND #1
11. 11: TS= Diabetes Mellitus
12. 12: TS= ((eye* or retina or retinal) NEAR/3 (screen* or exam* or check*))
13. 13: TS= (diabet* NEAR/2 eye NEAR/2 care)
14. 14: TS= (diabet* NEAR/2 eye NEAR/2 service*)
15. 15: #12 OR #13 OR #14
16. 16: #11 OR #9
17. 17: #15 AND #16
18. 18: #10 OR #17
19. 19: TI= (pregnan* or gestational)
20. 20: TI= ((deep or machine or transfer) NEAR/2 learning)
21. 21: TI= ((deep or convolutional or neural) NEAR/3 network*)
22. 22: TS= (retinopath* NEAR/2 prematurity)
23. 23: TS= (macular NEAR/2 degeneration)
24. 24: TI= (laser or photocoagulation or intravitreal or VEGF or bevacizumab or injection or dexamethasone or neuropathy or nephropathy or vascular or microvascular or neuropathy or nephropathy or vascular or microvascular)
25. 25: TI=(animal or primate or rat or rats or mouse or mice or pig)
26. 26: #25 OR #24 OR #23 OR #22 OR #21 OR #20 OR #19
27. 27: #18 NOT #26
28. 28: #18 NOT #26 and 2024 or 2023 or 2022 or 2021 or 2020 or 2019 or 2018 or 2017 or 2016 or 2015 or 2014 or 2008 or 2007 or 2006 or 2005 or 2004 or 2003 or 2002 or 2001 or 2000 (Publication Years)
