# Supplementary material for: What is the coverage of retina screening services for people with diabetes? Protocol for a systematic review and meta-analysis
Source: BMJ Open. 2024 Jan 30;14(1):e081123. doi: 10.1136/bmjopen-2023-081123 (PMC10828834; doi:10.1136/bmjopen-2023-081123)
Supplement: Supplementary data [file bmjopen-2023-081123supp003.pdf]

## Annex 3: Keywords for Google Search

Google Search Engine

Searches "All results" – first 10 pages, representing 1000 results to be screened

Combination of one of

1. Government
2. Health department
3. Ministry of health
4. Provincial health department
5. State health department

AND one of:

1. Afghanistan
2. Albania
3. Algeria
4. Andorra
5. Angola
6. Antigua and Barbuda
7. Argentina
8. Armenia
9. Australia
10. Austria
11. Azerbaijan
12. Bahamas
13. Bahrain
14. Bangladesh
15. Barbados
16. Belarus
17. Belgium
18. Belize
19. Benin
20. Bhutan
21. Bolivia
22. Bosnia and Herzegovina
23. Botswana
24. Brazil
25. Brunei Darussalam
26. Bulgaria
27. Burkina Faso
28. Burundi
29. Cabo Verde
30. Cambodia
31. Cameroon
32. Canada
33. Central African Republic
34. Chad
35. Chile
36. China
37. Colombia
38. Comoros
39. Congo
40. Cook Islands
41. Costa Rica
42. Cote d'Ivoire
43. Croatia
44. Cuba
45. Cyprus
46. Czechia
47. Democratic People's Republic of Korea
48. Democratic Republic of Congo
49. Denmark
50. Djibouti
51. Dominica
52. Dominican Republic
53. Ecuador
54. Egypt
55. El Salvador
56. Equatorial Guinea
57. Eritrea
58. Estonia
59. Eswatini
60. Ethiopia
61. Fiji
62. Finland
63. France
64. Gabon
65. Gambia
66. Georgia
67. Germany
68. Ghana
69. Greece
70. Grenada
71. Guatemala
72. Guinea
73. Guinea-Bissau
74. Guyana
75. Haiti
76. Honduras
77. Hungary
78. Iceland
79. India
80. Indonesia
81. Iran
82. Iraq
83. Ireland
84. Israel
85. Italy
86. Jamaica
87. Japan
88. Jordan
89. Kazakhstan
90. Kenya
91. Kiribati
92. Kuwait
93. Kyrgyzstan
94. Lao People's Democratic Republic
95. Latvia
96. Lebanon
97. Lesotho
98. Liberia
99. Libya
100. Lithuania
101. Luxembourg
102. Madagascar
103. Malawi
104. Malaysia
105. Maldives
106. Mali
107. Malta
108. Marshall Islands
109. Mauritania
110. Mauritius
111. Mexico
112. Federated States of Micronesia
113. Monaco
114. Mongolia
115. Montenegro
116. Morocco
117. Mozambique
118. Myanmar
119. Namibia
120. Nauru
121. Nepal
122. Netherlands
123. New Zealand
124. Nicaragua
125. Niger
126. Nigeria
127. Niue
128. North Macedonia
129. Norway
130. Oman
131. Pakistan
132. Palau
133. Panama
134. Papua New Guinea
135. Paraguay
136. Peru
137. Philippines
138. Poland
139. Portugal
140. Qatar
141. Republic of Korea
142. Republic of Moldova
143. Romania
144. Russian Federation

145. Rwanda
146. Saint Kitts and Nevis
147. Saint Lucia
148. Saint Vincent and the Grenadines
149. Samoa
150. San Marino
151. Sao Tome and Principe
152. Saudi Arabia
153. Senegal
154. Serbia
155. Seychelles
156. Sierra Leone
157. Singapore
158. Slovakia
159. Slovenia
160. Solomon Islands
161. Somalia
162. South Africa
163. South Sudan
164. Spain
165. Sri Lanka
166. Sudan
167. Suriname
168. Sweden
169. Switzerland
170. Syrian Arab Republic
171. Tajikistan
172. Thailand
173. Timor-Leste
174. Togo
175. Tonga
176. Trinidad and Tobago
177. Tunisia
178. Türkiye
179. Turkmenistan
180. Tuvalu
181. Uganda
182. Ukraine
183. United Arab Emirates
184. United Kingdom
185. United Republic of Tanzania
186. United States of America
187. Uruguay
188. Uzbekistan
189. Vanuatu
190. Venezuela
191. Vietnam
192. Yemen
193. Zambia
194. Zimbabwe
195. American Samoa
196. Bermuda
197. French Polynesia
198. Greenland
199. Hong Kong
200. Palestine
201. Puerto Rico
202. Tokelau
