# Supplementary material for: What is the coverage of retina screening services for people with diabetes? Protocol for a systematic review and meta-analysis
Source: BMJ Open. 2024 Jan 30;14(1):e081123. doi: 10.1136/bmjopen-2023-081123 (PMC10828834; doi:10.1136/bmjopen-2023-081123)
Supplement: Supplementary data [file bmjopen-2023-081123supp004.pdf]

Annex 4: Keywords for individual website searches

1. Diabetic eye screening
2. Diabetic retinopathy screening
3. Diabetic retinopathy screening AND coverage
4. Retina screening coverage
5. Retina screening AND adherence
6. Retina screening AND uptake
