# Supplementary material for: What is the coverage of retina screening services for people with diabetes? Protocol for a systematic review and meta-analysis
Source: BMJ Open. 2024 Jan 30;14(1):e081123. doi: 10.1136/bmjopen-2023-081123 (PMC10828834; doi:10.1136/bmjopen-2023-081123)
Supplement: Supplementary data [file bmjopen-2023-081123supp005.pdf]

BMJ Publishing Group Limited (BMJ) disclaims all liability and responsibility arising from any reliance placed on this supplemental material which has been supplied by the author(s)

Annex 5: Decision tree for inclusion of retina screening coverage for people with diabetes data presented per country for the preferred and alternative data sources for the WHO Definition (shown in Box 1)

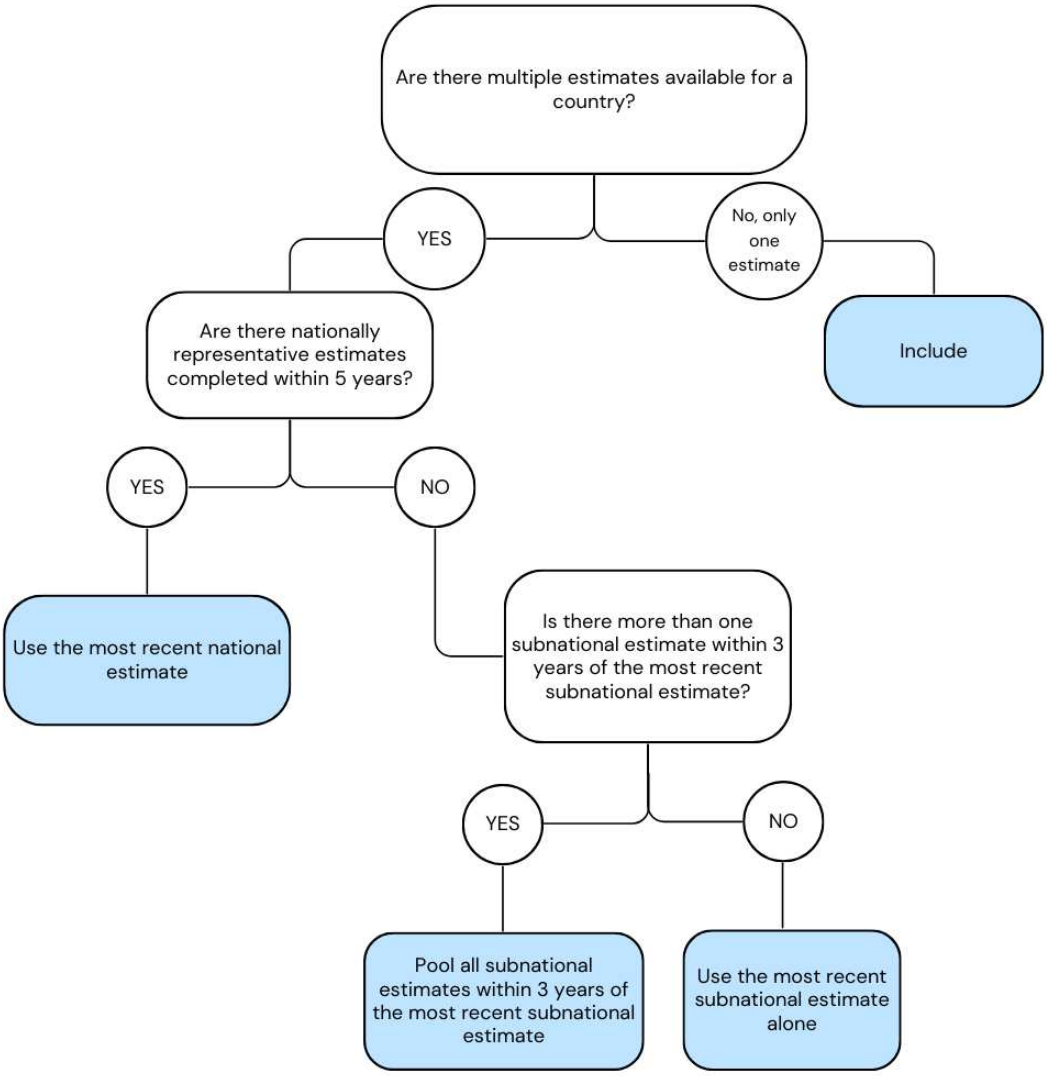

Chabba N, *et al. BMJ Open* 2024; 14:e081123. doi: 10.1136/bmjopen-2023-081123
