# Supplementary material for: What is the coverage of retina screening services for people with diabetes? Protocol for a systematic review and meta-analysis
Source: BMJ Open. 2024 Jan 30;14(1):e081123. doi: 10.1136/bmjopen-2023-081123 (PMC10828834; doi:10.1136/bmjopen-2023-081123)

BMJ Open is committed to open peer review. As part of this commitment we make the peer review history of every article we publish publicly available.

When an article is published we post the peer reviewers' comments and the authors' responses online. We also post the versions of the paper that were used during peer review. These are the versions that the peer review comments apply to.

The versions of the paper that follow are the versions that were submitted during the peer review process. They are not the versions of record or the final published versions. They should not be cited or distributed as the published version of this manuscript.

BMJ Open is an open access journal and the full, final, typeset and author-corrected version of record of the manuscript is available on our site with no access controls, subscription charges or pay-per-view fees (http://bmjopen.bmj.com).

If you have any questions on BMJ Open's open peer review process please email info.bmjopen@bmj.com

BMJ Open

What is the coverage of retina screening services for people with diabetes? Protocol for a systematic review and meta-analysis

Journal:	*BMJ Open*
Manuscript ID	bmjopen-2023-081123
Article Type:	Protocol
Date Submitted by the Author:	19-Oct-2023

Complete List of Authors:	Chabba, Nimisha; University of Auckland<br>Silwal, Pushkar; University of Auckland<br>Bascaran, Covadonga ; London School of Hygiene and Tropical Medicine International Centre for Eye Health,<br>McCormick, Ian; London School of Hygiene and Tropical Medicine, International Centre for Eye Health<br>Goodman, Lucy; University of Auckland<br>Gordon, Iris; London School of Hygiene & Tropical Medicine, International Centre for Eye Health<br>Burton, Matthew J; London School of Hygiene & Tropical Medicine, International Centre for Eye Health<br>Keel, Stuart ; World Health Organization, Department of Noncommunicable Disease<br>Evans, Jennifer; London School of Hygiene & Tropical Medicine, International Centre for Eye Health<br>Ramke, Jacqueline; University of Auckland; London School of Hygiene & Tropical Medicine, International Centre for Eye Health

Keywords:	Diabetic retinopathy < DIABETES & ENDOCRINOLOGY, Mass Screening, General diabetes < DIABETES & ENDOCRINOLOGY

SCHOLARONE™ Manuscripts

What is the coverage of retina screening services for people with diabetes? Protocol for a systematic review and meta-analysis

Nimisha Chabba[1]

Pushkar Silwal[1]

Covadonga Bascaran[2]

Ian McCormick[2]

Lucy Goodman[1]

Iris Gordon[2]

Matthew J Burton[2,3]

Stuart Keel[4]

Jennifer R Evans[2]

Jacqueline Ramke[1,2]

1. School of Optometry and Vision Science, University of Auckland, Auckland, New Zealand
2. International Centre for Eye Health, London School of Hygiene & Tropical Medicine, London, UK
3. National Institute for Health Research Biomedical Research Centre for Ophthalmology at Moorfields Eye Hospital NHS Foundation Trust and UCL Institute of Ophthalmology, London, UK
4. Department of Noncommunicable Disease, World Health Organization, Geneva, Switzerland

Corresponding Author:

Nimisha Chabba

School of Optometry and Vision Science, University of Auckland, Auckland, New Zealand

nimisha.chabba@auckland.ac.nz

For peer review only

Abstract

Introduction

Diabetic retinopathy is a leading cause of vision impairment globally. Vision loss from diabetic retinopathy can generally be prevented by early detection and timely treatment. The World Health Organization (WHO) included a measure of service access for diabetic retinopathy as a core indicator in the Eye Care Indicator Menu launched in 2022: *Retina screening coverage for people with diabetes*. The aim of this review is to provide a comprehensive global and regional summary of the available information on retina screening coverage for people with diabetes.

Methods and analysis

A search will be conducted in Medline (Ovid) and Embase without language restrictions for studies from any country reporting retina screening coverage for adults with any type of diabetes at the national or subnational level using data collected since 1 January 2000. We will also seek reports and coverage statistics from government websites of all WHO member states. Two investigators will independently screen studies, extract relevant data and assess risk of bias of included studies. The review will be reported using the Preferred Reporting Items for Systematic Review and Meta-Analysis guideline. We will summarise the range of coverage definitions reported across included studies and present the median retina screening coverage in WHO regions and by World Bank country income level. Depending on the availability of data, we will conduct meta-analysis to assess disparities in retina screening coverage for people with diabetes by factors in the PROGRESS framework (Place of residence, Race/ethnicity/culture/language, Occupation, Gender/sex, Religion, Education, Socioeconomic status, and Social capital).

Ethics and dissemination

This review will only include published data thus no ethical approval will be sought. The findings of this review will be published in a peer-reviewed journal and presented at relevant conferences.

Registration

OSF registration 17/10/2023: https://osf.io/k5p69

Strengths and limitations of this study

- A strength of this review is the comprehensive search, designed by an experienced information specialist, and without any language or geographical restrictions, across two databases for reports published from 1 January 2000.
- In addition, an extensive and systematic search of government websites (grey literature) will be undertaken and field experts will be requested to nominate additional potentially relevant reports.
- A further strength is that study selection, data extraction and quality assessment will be conducted by two investigators independently.
- A potential limitation is that anticipated heterogeneity in the definitions of the outcome measure, reporting period, and method of data collection may preclude quantitative synthesis of results.

For peer review only - http://bmjopen.bmj.com/site/about/guidelines.xhtml

Introduction

Diabetic retinopathy is a leading cause of vision impairment globally, and the only one of the five leading causes that increased in prevalence in the last 30 years (1). Most of the vision impairment caused by diabetic retinopathy is avoidable through early detection and timely treatment (2, 3). However, vision that has already been lost due to diabetic retinopathy cannot be restored (4). Many countries have retina screening programmes for diabetic retinopathy (also called diabetic eye screening or diabetic retinopathy screening) that are administered at a national-, regional- or facility-level and aim to identify all patients with sight-threatening retinopathy, with subsequent treatment by the same service or via referral to (generally co-located) treatment services (5).

Diabetic retinopathy screening programmes will only be successful in reducing vision loss if most people with diabetes are routinely screened and referred for treatment where indicated. The recommended screening interval varies between countries (e.g., one year in Kenya, 1-2 years in England and 2-3 years in New Zealand) (5, 6). Regardless of the recommended period, available evidence confirms that attendance at retinal screening is suboptimal, with services being less accessible for some population groups (7, 8). People who experience delayed or reduced access to screening will be less likely to be offered timely initiation of treatment should they require it (9).

A measure for diabetic retinopathy service access: *Retina screening coverage for people with diabetes* was recently proposed by the World Health Organization (WHO) as a core indicator for eye health (10). This indicator uses routinely collected health facility data or population-based surveys to measure the percentage of people with known/ diagnosed diabetes who undertake a retina examination at the interval recommended and defined in nationally adopted guidelines. In countries without a recommended screening interval, WHO recommends screening every two years. The way that studies define or report coverage varies. For example, while some studies report coverage at a specific point in time from cross-sectional facility-based data (11) or self-reported service use in survey data (12), others report the proportion of the enrolled diabetic population who attend all expected screening events within a longer period of time (e.g. 5 years of follow-up (13, 14)).

Measuring retina screening coverage is essential to help monitor progress towards the prevention of sight-threatening diabetic retinopathy (15). WHO recently included this indicator in the District Health Information Systems, version 2 (DHIS2) to support the integration and systematic collection of eye care data into facility level reporting systems (16). To date, retina screening coverage for people with diabetes has not been summarised systematically at the global or regional level.

The aim of this review is to provide a comprehensive global and regional summary of the available information on retina screening coverage for people with diabetes.

Objectives

The objectives of this review are to:

(1) Summarise the different ways retina screening coverage for people with diabetes has been defined in published reports.
(2) Summarise retina screening coverage for people with diabetes by World Bank country income level and WHO regions.
(3) Compare disparities in retina screening coverage for people with diabetes by factors in the PROGRESS framework (i.e., between subgroups within any of: Place of residence, Race/ethnicity/culture/language, Occupation, Gender/sex, Religion, Education, Socioeconomic status, and Social capital) (17).

For peer review only - http://bmjopen.bmj.com/site/about/guidelines.xhtml

Methods and analysis

This protocol is reported in accordance with the Preferred Reporting Items for Systematic Reviews and Meta-Analysis Protocols (PRISMA-P) checklist (18, 19). The completed PRISMA-P checklist is presented in Annex 1. The protocol has been registered with the Open Science Framework registry and can be viewed at: https://osf.io/k5p69.

Eligibility criteria

Population and Context

We will include reports from any country reporting retina screening coverage for adults for any type of diabetes. There will be no restrictions on patient characteristics such as sex, ethnicity, duration of diabetes or location. We will exclude studies reporting outcomes only in children (aged 18 years or below) due to the higher likelihood of people <18 years having Type 1 diabetes, with a delayed first screening recommendation. We will exclude studies reporting outcomes only for people with pre-diabetes, and studies reporting outcomes only for people with gestational diabetes because it is mainly a temporary type of diabetes with different screening recommendations.

Type of studies

- Type of report: We will include published (peer reviewed) literature where the full report is published i.e., we will exclude reports with only an abstract available, as well as editorials, protocols and reports of pilot studies. We will also exclude systematic reviews or other evidence syntheses but will examine the reference list of relevant reviews to identify potentially relevant primary studies. We will include grey literature from governments that meet our other inclusion criteria.
- Source of data: We will include data derived from the following sources:
    - population-based research studies;
    - facility-based data collected routinely or *de novo;*
    - service activity or payment data from Ministries of Health or health insurance databases.

  These sources of data may be reported at the national or subnational (state/province/district) level. We will exclude studies reporting data from a small number of facilities unless we consider the sampling representative of the national or state/province/district level (e.g. in small island states).
- Study design: We will include cross-sectional studies, cohort studies, randomised controlled trials (for the control arm[s]) and non-randomised intervention studies (for the control arm[s]). We will exclude studies only reporting modelled data.
- Other: Due to the changing nature of the health system over time, we will limit our review to data collected since 1 January 2000. We will exclude studies reporting data exclusively during the peak of the COVID-19 pandemic (2020 and 2021). If an included study reports estimates from both COVID-19 and non-COVID-19 years, we will include data from the non-COVID-19 period. Our search will have no language restrictions and every effort will be made to translate reports in languages other than English.

Outcome

We will include studies that report a measure of retina screening coverage that includes a numerator and denominator value at a national, or state/province/district level, regardless of the screening interval used (e.g., annual, biennial). We will also include studies that report the number of people with diabetes who attend all of their expected retina screening appointments during a defined follow-up period. The screening could involve a retinal examination by a relevant health practitioner or where an attempt is made to image the retina of a patient by digital photography to determine the presence (and grading) of diabetic retinopathy by a human grader and/or by artificial intelligence. We will exclude studies reporting a coverage proportion without also reporting at least one of either the numerator or denominator and a defined screening interval or time period. We will also exclude studies only reporting attendance at appointments subsequent to retinal screening (e.g., referral to an eye clinic for further assessment).

When a potentially eligible study only reports coverage disaggregated by population groups and not overall, we will cross-check our included studies to determine if the overall coverage proportion is available in a separate

For peer review only - http://bmjopen.bmj.com/site/about/guidelines.xhtml

publication; in the event it is not, we will contact study authors to request the overall coverage. If the overall coverage can be provided, we will include the study. In the event the overall coverage cannot be provided, we will include the study only if the population groups for which the data are disaggregated are relevant to the PROGRESS framework, and this study will be considered for analysis of objective 1 and 3 but not objective 2. For example, we will include a study disaggregating coverage for immigrants and non-immigrants, but not a study disaggregating coverage for people with different levels of vision difficulty.

Information sources and search strategy

We will search MEDLINE and Embase for reports using data collected since 1 January 2000 using a search strategy developed by an experienced Information Specialist (IG). Our MEDLINE search strategy is included in Annex 2. We will download and de-duplicate the results in EndNote, and then export the results into Covidence (Veritas Health Innovation, Melbourne, Australia. Available at www.covidence.org) for screening. We will examine reference lists of all included reports to identify further potentially relevant reports.

We will search for grey literature in the form of government reports of retina screening coverage at the national or subnational level. Grey literature reports will be searched for by one investigator (NC); another investigator (LG) will repeat the searches for 20 countries to ensure the consistency of relevant results. Government websites, including that of national and provincial government health departments, of the 194 World Health Organization member states and eight associate countries and territories (American Samoa, Bermuda, French Polynesia, Greenland, Hong Kong, Palestine, Puerto Rico, and Tokelau) will be identified and searched for relevant reports and coverage statistics. The process for systematically collecting government reports will be based on the grey literature search framework outlined by Godin et al (20). First, to identify the relevant government websites, a Google search will be conducted using the search strategy in Annex 3. The first ten pages of each search (representing 100 results) will be reviewed, using the title and short text underneath. The relevant websites' name/organisation, country and URL will be entered into an Excel spreadsheet. Then, each of these websites will be searched using the search feature and a combination of keywords included in Annex 4; when the search feature is not available, the website will be scanned for relevant information. When the search feature is available, results will be reviewed using the title and short text underneath and until no more relevant items are identified on a following page. All potentially relevant reports will be exported into Covidence and de-duplicated for title and abstract screening.

Selection

For published literature and grey literature, all title and abstracts (for published literature) or short text (for grey literature) will be screened by two investigators independently using Covidence systematic review management software (Veritas Health Innovation, Melbourne, Australia; available at www.covidence.org). Full-texts of all potentially relevant publications will then be acquired and assessed by two investigators independently to establish eligibility for inclusion into the review. Any disagreements in the screening of search results will be resolved by discussion and consultation with a third investigator as needed.

Following the selection process of published and grey literature, field experts—including clinicians, academics, non-governmental staff, the Diabetic Retinopathy Work Group of the International Agency for the Prevention of Blindness (IAPB) and WHO representatives—will be provided with a list of the included reports and requested to nominate further potentially relevant reports. An Excel spreadsheet will be used to track who has been contacted, who has responded, the need for a follow-up message, and identified reports.

Data collection and data items

Data extraction will be performed in Covidence. The data extraction form will be piloted by two investigators on five studies and modifications undertaken as required. The following details of each report will be extracted by two investigators independently. Any discrepancies will be resolved by discussion, and with a third reviewer if necessary. In the event of unclear outcome data, two attempts will be made to contact the corresponding author on email.

For peer review only - http://bmjopen.bmj.com/site/about/guidelines.xhtml

- ***Study characteristics***: country/countries of study, type of report, source of data, study design, administrative region of study (when sub-national e.g., province, district, state), year(s) of data collection, number of participants, overall participation rate, extent of missing data.
- ***Participant characteristics***: inclusion criteria (age, diabetes type), age (median/mean and range), diabetes type, diabetic retinopathy status, vision impairment status (21), available information across any PROGRESS dimension (outlined above) (17).
- ***Outcome(s)***: definition of screening coverage (numerator, denominator, screening interval), period of follow-up (if applicable), retina screening coverage result (aggregated and if disaggregated by diabetes type, diabetic retinopathy status or any equity/PROGRESS dimensions (outlined above) (17)).

Outcomes

The primary outcome measure is retina screening coverage for people with diabetes. This is defined in a range of ways. We will include the following broad categories of outcomes and will expand this list based on what is identified during the search.

Box 1: Anticipated definitions of retina screening coverage for people with diabetes

**Definition category** (Reference/example)	**Details**	
WHO definition and data sources (10)	**Data source 1: Preferred data source = facility**	***Definition***: the proportion of people with diabetes who undertook a retina examination at the recommended interval.<br>***Numerator***= Number of people with diabetes who undertook a retina examination at the recommended interval.<br>***Denominator***= Total number of people registered in a facility or, if a register is not available, the estimated prevalence of diabetes in the population covered by a facility.<br>***Interval***= As defined in national screening guidelines or biennially if not outlined in a guideline.
	**Data source 2: Alternative data source = population-based surveys***	***Numerator***= Number of people in the survey who self-report attendance at an eye check for diabetes.<br>***Denominator***= Number of people in the survey with diabetes (either self-reported or confirmed with testing).<br>***Interval***= Either not specified (e.g., the participant is asked when they last attended a check) or a specified period (e.g., the participant is asked whether they attended a check within the last 12 months / 2 years / other time period).
Other definitions	Cohort from facility-based data (14)	***Numerator***= Number of people in the cohort who had attended all of the expected retinal screening appointments (as per the national guideline or biennially) during the follow-up period.<br>***Denominator***= Total number of people registered with diabetes OR if that is not available, the number of people with diabetes who had at least one retinal screening appointment during the follow-up period.<br>***Interval***= As defined in national screening guidelines or biennially if not outlined in a guideline.
	Attendance following diabetes diagnosis (22)	***Numerator***= Number of people with newly diagnosed diabetes who attended the first retinal screening appointment.<br>***Denominator***= Total number of people registered with newly diagnosed diabetes.<br>***Interval***= Within a specified period e.g., within 12 months of diabetes diagnosis.

*The WHO definition requires facility data. For this alternative data source, we have proposed this definition and will reflect on variations of it in reporting of objective 1.

Risk of bias in individual studies

Two investigators will independently assess the risk of bias in each included study. Any conflict in relation to the appraisal will be discussed between the two investigators and resolved with a third investigator if necessary.

For peer review only - http://bmjopen.bmj.com/site/about/guidelines.xhtml

Relevant Joanna Briggs Institute (JBI) critical appraisal tools will be used to evaluate each included study (including tools for cohort studies and prevalence studies) (23).

Assessment of heterogeneity

Clinical heterogeneity will be assessed by comparing key participant characteristics at the study level (e.g., age, sex, diabetes type, diabetic retinopathy status). Methodological heterogeneity will also be considered, including variation in the specific definition of the numerator and denominator as well as the reporting period and method of data collection. If meta-analysis is possible, we will assess statistical heterogeneity by inspecting forest plots and the I^2 and τ^2 statistics to examine the proportion of heterogeneity across studies that is due to chance. If high levels of heterogeneity are detected (for example I^2 >50%) and there are sufficient data available (for example, meta-analysis including more than 10 studies), we will explore likely sources of this heterogeneity via meta-regression.

Data synthesis

We will take an inclusive approach for objective 1 and will then determine the potential for synthesis of results reporting the WHO definition (Box 1) for objective 2, before exploring disparities within all included studies in objective 3.

To achieve objective 1, we will outline the full range of definitions used across included studies and summarise the study characteristics of each definition, including geographic (country, national vs sub-national), source of data, study design, inclusion criteria and extent of data missingness.

To achieve objective 2, in countries where estimates on retina screening coverage for people with diabetes using the WHO definition are available from multiple sources, we will establish the country estimate according to the decision tree included in Annex 5. We will first follow the process separately for estimates generated for the preferred and alternative data sources for the WHO definition (facility- and survey-based respectively, see Box 1). The process outlined in the decision tree prioritises more recent studies and studies with nationally representative sampling frames. Where no nationally representative estimates are available, we will pool two or more subnational estimates if the definitions of the outcome are comparable, and the estimates are close enough in value that the pooled result provides a meaningful measure of retina screening coverage for people with diabetes. We will combine these subnational estimates from the same country using an inverse variance weighted average, using the metagen command from the meta package in R. If two sources of data are then available for a country, we will prioritise facility data in countries with organised retina screening programmes and accurate diabetes registries, and survey data in countries with opportunistic screening or with no centralised registries. We will document the extent to which these judgements affect the final country estimates by a sensitivity analysis.

We will use the country estimates to calculate the median, interquartile range (IQR) and range of retina screening coverage for each WHO region and World Bank country income level (24), stratified by screening interval (e.g., 1- 2- and 3-years). We will also compare retina screening coverage estimates over time where comparable sampling frames have been used in repeat studies.

To achieve our third objective, and depending on availability of data, we will calculate risk ratios comparing coverage in different groups, specifically:

- diabetes type (type 2 versus type 1)
- duration of diabetes (per year increase)
- diabetic retinopathy status (present versus absent)

and PROGRESS categories:

- place of residence (e.g., rural versus urban)
- ethnicity (e.g., non-dominant versus dominant)
- occupation status (e.g., not-employed versus employed).
- gender/sex (e.g., female versus male)

For peer review only - http://bmjopen.bmj.com/site/about/guidelines.xhtml

- religion (e.g., non-dominant versus dominant)
- education level (e.g., no educational qualification versus school level qualification versus further educational qualification)
- socioeconomic status (e.g., high versus low area level deprivation)
- social capital (e.g., low versus high social capital)

We will plot these risk ratios in separate forest plots and combine values where appropriate i.e., where we judge that pooling will provide a meaningful measure of comparative risk (see assessment of heterogeneity section above), using the metagen command from the meta package in R. If there are sufficient data available (for example, meta-analysis including 10 or more studies), we will also assess small study effects, one of which may be publication bias, by preparing a funnel plot (25), which is a scatter plot of effect size versus precision (standard error).

Certainty assessment

Currently there is no formal guidance from GRADE regarding the assessment of certainty in systematic reviews and meta-analysis of prevalence studies (26). Therefore, we do not plan to assess certainty formally but we will consider the GRADE domains i.e. risk of bias, inconsistency, imprecision, indirectness and publication bias when drawing conclusions from the available evidence (27).

Patient and public involvement

Patients and the public will not be involved in the conduct of the systematic review and meta-analysis, as we plan to review existing published literature and publicly available reports only.

Ethics and dissemination

This review will only include published data thus no ethical approval will be sought. The findings of this review will be published in a peer-reviewed journal and presented at relevant conferences.

For peer review only

For peer review only - http://bmjopen.bmj.com/site/about/guidelines.xhtml

References

(1) Steinmetz JD, Bourne RRA, Briant PS, Flaxman SR, Taylor HRB, Jonas JB, et al. Causes of blindness and vision impairment in 2020 and trends over 30 years, and prevalence of avoidable blindness in relation to VISION 2020: the Right to Sight: an analysis for the Global Burden of Disease Study. The Lancet Global Health. 2021;9(2):e144–60.

(2) Burton MJ, Ramke J, Marques AP, Bourne RRA, Congdon N, Jones I, et al. The Lancet Global Health Commission on Global Eye Health: vision beyond 2020. The Lancet Global Health. 2021;9(4):e489–551.

(3) Vujosevic S, Aldington SJ, Silva P, Hernández C, Scanlon P, Peto T, et al. Screening for diabetic retinopathy: new perspectives and challenges. The Lancet Diabetes & Endocrinology. 2020;8(4):337–47.

(4) The International Agency for the Prevention of Blindness. Diabetic Retinopathy. 2023. https://www.iapb.org/learn/resources/diabetic-retinopathy/ (accessed 18 June 2023).

(5) Das T, Takkar B, Sivaprasad S, Thanksphon T, Taylor H, Wiedemann P, Nemeth J, Nayar PD, Rani PK, Khandekar R. Recently updated global diabetic retinopathy screening guidelines: commonalities, differences, and future possibilities. Eye. 2021;35(10):2685-98.

(6) The College of Optometrists. Changes to the NHS Diabetic Eye Screening Programme in England. The College of Optometrists. https://www.college-optometrists.org/news/2023/october/changes-nhs-diabetic-eye-screening-programme (accessed 14 October 2023)

(7) Wong TY, Sabanayagam C. Strategies to tackle the global burden of diabetic retinopathy: from epidemiology to artificial intelligence. Ophthalmologica. 2020;243(1):9-20.

(8) Silwal PR, Lee AC, Squirrell D, Zhao J, Harwood M, Vincent AL, Murphy R, Ameratunga S, Ramke J. Use of public sector diabetes eye services in New Zealand 2006–2019: Analysis of national routinely collected datasets. Plos one. 2023;18(5):e0285904.

(9) Ting DS, Cheung GC, Wong TY. Diabetic retinopathy: global prevalence, major risk factors, screening practices and public health challenges: a review. Clinical & experimental ophthalmology. 2016;44(4):260-77.

(10) World Health Organization. Eye care indicator menu (ECIM): a tool for monitoring strategies and actions for eye care provision. Geneva: World Health Organization; 2022.

(11) Wilson A, Baker R, Thompson J, Grimshaw G. Coverage in screening for diabetic retinopathy according to screening provision: results from a national survey in England and Wales. Diabetic medicine. 2004;21(3):271-8.

(12) Foreman J, Keel S, Xie J, Van Wijngaarden P, Taylor HR, Dirani M. Adherence to diabetic eye examination guidelines in Australia: the National Eye Health Survey. Medical Journal of Australia. 2017;206(9):402-6.

(13) Gibson AA, Humphries J, Gillies M, Nassar N, Colagiuri S. Adherence to eye examination guidelines among individuals with diabetes: an analysis of linked health data. Clinical & Experimental Ophthalmology. 2020;48(9):1229-38.

(14) An J, Niu F, Turpcu A, Rajput Y, Cheetham TC. Adherence to the American Diabetes Association retinal screening guidelines for population with diabetes in the United States. Ophthalmic epidemiology. 2018;25(3):257-65.

(15) World Health Organisation Regional Office for Europe. Diabetic retinopathy screening: a short guide. Increase effectiveness, maximise benefits and minimise harm. Copenhagen: WHO Regional Office for Europe, 2020.

(16) World Health Organization. Guidance on the analysis and use of routine health information systems: eye and ear care module. Geneva: World Health Organization; 2023.

(17) O'Neill J, Tabish H, Welch V, Petticrew M, Pottie K, Clarke M, Evans T, Pardo JP, Waters E, White H, Tugwell P. Applying an equity lens to interventions: using PROGRESS ensures consideration of socially stratifying factors to illuminate inequities in health. Journal of clinical epidemiology. 2014;67(1):56-64.

(18) Moher D, Shamseer L, Clarke M, Ghersi D, Liberati A, Petticrew M, Shekelle P, Stewart LA. Preferred Reporting Items for Systematic Review and Meta-Analysis Protocols (PRISMA-P) 2015 statement. Syst Rev. 2015;4(1):1.

(19) Shamseer L, Moher D, Clarke M, Ghersi D, Liberati A, Petticrew M, Shekelle P, Stewart LA, the PRISMA-P Group. Preferred Reporting Items for Systematic Review and Meta-Analysis Protocols (PRISMA-P) 2015: elaboration and explanation. BMJ 2015.349:g7647.

(20) Godin K, Stapleton J, Kirkpatrick SI, Hanning RM, Leatherdale ST. Applying systematic review search methods to the grey literature: a case study examining guidelines for school-based breakfast programs in Canada. Systematic reviews. 2015;4(1):1-0.

(21) World Health Organization. International Classification of Diseases. 11th rev., ICD-11. Geneva: World Health Organization, 2018.

(22) Scanlon PH, Stratton IM, Leese GP, Bachmann MO, Land M, Jones C, Ferguson B, Four Nations Diabetic Retinopathy Screening Study Group. Screening attendance, age group and diabetic retinopathy level at first screen. Diabetic Medicine. 2016;33(7):904-11.

(23) JBI. Critical Appraisal Tools. JBI. 2023. https://jbi.global/critical-appraisal-tools (accessed 20 July 2023).

(24) The World Bank. The world by income and region. 2023. https://datatopics.worldbank.org/world-development-indicators/the-world-by-income-and-region.html (accessed 15 August 2023).

(25) Egger M, Smith GD, Schneider M, Minder C. Bias in meta-analysis detected by a simple, graphical test. BMJ. 1997;315(7109):629-34.

For peer review only - http://bmjopen.bmj.com/site/about/guidelines.xhtml

(26) Borges Migliavaca C, Stein C, Colpani V, Barker TH, Munn Z, Falavigna M, Prevalence Estimates Reviews–Systematic Review Methodology Group (PERSyst). How are systematic reviews of prevalence conducted? A methodological study. BMC medical research methodology. 2020;20:1-9.

(27) Schünemann H. Brożek J. Guyatt G. Oxman A. editors. GRADE handbook for grading quality of evidence and strength of recommendations. Updated October 2013. The GRADE Working Group, 2013. Available from guidelinedevelopment.org/handbook.

For peer review only

For peer review only - http://bmjopen.bmj.com/site/about/guidelines.xhtml

Authors' contributions

JR conceived the idea for the review. NC and JR drafted and revised the protocol with suggestions from PS, CB, LG, IM, MJB, SK and JE. IG constructed the search.

Funding

NC is supported by a University of Auckland Doctoral Scholarship. PS is supported by a Buchanan Charitable Foundation Postdoctoral Fellowship. MJB is supported by the Wellcome Trust (207472/Z/17/Z).

Competing interests

None declared.

Word Count

2843

ORCID iDs

Nimisha Chabba https://orcid.org/0009-0009-2863-7322

Pushkar Silwal https://orcid.org/0000-0001-6100-090X

Covadonga Bascaran https://orcid.org/0000-0002-5662-3325

Ian McCormick https://orcid.org/0000-0002-7360-3844

Lucy Goodman https://orcid.org/0000-0002-3533-7717

Iris Gordon https://orcid.org/0000-0001-8143-8132

Matthew J Burton https://orcid.org/0000-0003-1872-9169

Stuart Keel https://orcid.org/0000-0001-6756-348X

Jennifer R Evans https://orcid.org/0000-0002-6137-2030

Jacqueline Ramke http://orcid.org/0000-0002-5764-1306

For peer review only

For peer review only - http://bmjopen.bmj.com/site/about/guidelines.xhtml

Annex 1: Reporting of PRISMA-P 2015 Items

**Section and topic**	**Item No**	**Checklist item**	**Location where reported**
**ADMINISTRATIVE INFORMATION**			
Title:			
Identification	1a	Identify the report as a protocol of a systematic review	1
Update	1b	If the protocol is for an update of a previous systematic review, identify as such	N/A
Registration	2	If registered, provide the name of the registry (such as PROSPERO) and registration number	1
Authors:			
Contact	3a	Provide name, institutional affiliation, e-mail address of all protocol authors; provide physical mailing address of corresponding author	1
Contributions	3b	Describe contributions of protocol authors and identify the guarantor of the review	11
Amendments	4	If the protocol represents an amendment of a previously completed or published protocol, identify as such and list changes; otherwise, state plan for documenting important protocol amendments	N/A
Support:			
Sources	5a	Indicate sources of financial or other support for the review	11
Sponsor	5b	Provide name for the review funder and/or sponsor	11
Role of sponsor or funder	5c	Describe roles of funder(s), sponsor(s), and/or institution(s), if any, in developing the protocol	N/A
**INTRODUCTION**			
Rationale	6	Describe the rationale for the review in the context of what is already known	3
Objectives	7	Provide an explicit statement of the question(s) the review will address with reference to participants, interventions, comparators, and outcomes (PICO)	3
**METHODS**			
Eligibility criteria	8	Specify the study characteristics (such as PICO, study design, setting, time frame) and report characteristics (such as years considered, language, publication status) to be used as criteria for eligibility for the review	4
Information sources	9	Describe all intended information sources (such as electronic databases, contact with study authors, trial registers or other grey literature sources) with planned dates of coverage	5
Search strategy	10	Present draft of search strategy to be used for at least one electronic database, including planned limits, such that it could be repeated	Annex 2-4
Study records:			
Data management	11a	Describe the mechanism(s) that will be used to manage records and data throughout the review	5
Selection process	11b	State the process that will be used for selecting studies (such as two independent reviewers) through each phase of the review (that is, screening, eligibility and inclusion in meta-analysis)	5
Data collection process	11c	Describe planned method of extracting data from reports (such as piloting forms, done independently, in duplicate), any processes for obtaining and confirming data from investigators	5-6
Data items	12	List and define all variables for which data will be sought (such as PICO items, funding sources), any pre-planned data assumptions and simplifications	5-6
Outcomes and prioritization	13	List and define all outcomes for which data will be sought, including prioritization of main and additional outcomes, with rationale	5-6
Risk of bias in individual studies	14	Describe anticipated methods for assessing risk of bias of individual studies, including whether this will be done at the outcome or study level, or both; state how this information will be used in data synthesis	6
Data synthesis	15a	Describe criteria under which study data will be quantitatively synthesised	6-7
	15b	If data are appropriate for quantitative synthesis, describe planned summary measures, methods of handling data and methods of combining data from studies, including any planned exploration of consistency (such as I^2, Kendall's τ)	6-7
	15c	Describe any proposed additional analyses (such as sensitivity or subgroup analyses, meta-regression)	6-7
	15d	If quantitative synthesis is not appropriate, describe the type of summary planned	7
Meta-bias(es)	16	Specify any planned assessment of meta-bias(es) (such as publication bias across studies, selective reporting within studies)	7-8
Confidence in cumulative evidence	17	Describe how the strength of the body of evidence will be assessed (such as GRADE)	8

For peer review only - http://bmjopen.bmj.com/site/about/guidelines.xhtml

Annex 2: Search Strategy

Database: Ovid MEDLINE(R) ALL <1946 to September 20, 2023>

----

1 exp Diabetes Mellitus/ (510992)
2 exp Diabetes Complications/ (150270)
3 Diabetic Retinopathy/ (29738)
4 ((diabet$ or proliferative or non-proliferative) adj4 retinopath$).tw. (32766)
5 diabetic retinopathy.kw. (5910)
6 (diabet$ adj3 (eye$ or vision or visual$ or sight$)).tw. (5839)
7 (retinopath$ adj3 (eye$ or vision or visual$ or sight$)).tw. (2635)
8 (DR adj3 (eye$ or vision or visual$ or sight$)).tw. (1135)
9 (diabet$ adj3 macula$ adj3 oedema).tw. (929)
10 (diabet$ adj3 macula$ adj3 edema).tw. (4896)
11 (DMO or DME).tw. (5650)
12 or/1-11 (526533)
13 Vision Screening/ (2471)
14 12 and 13 (300)
15 Mass Screening/ (116536)
16 or/3-11 (48924)
17 15 and 16 (1230)
18 14 or 17 (1511)
19 ((eye$ or retina or retinal) adj3 (screen$ or exam$ or check$)).tw. (21366)
20 (diabet$ adj2 eye adj2 care).tw. (101)
21 (diabet$ adj2 eye adj2 service$).tw. (23)
22 or/19-21 (21426)
23 12 and 22 (3021)
24 18 or 23 (4027)
25 (pregnan$ or gestational).ti. (291008)
26 artificial intelligence/ (40249)
27 deep learning/ (16593)
28 exp machine learning/ (60204)
29 "neural networks (computer)"/ (48862)
30 fuzzy logic/ (5319)
31 (artificial adj1 intelligence).ti. (14681)
32 ((deep or machine or transfer) adj2 learning).ti. (56299)
33 ((deep or convolutional or neural) adj3 network$).ti. (35779)
34 or/25-33 (468443)
35 24 not 34 (3796)
36 case reports/ (2357424)
37 (case adj3 report$).tw. (702606)
38 or/36-37 (2498698)
39 35 not 38 (3636)
40 (retinopath$ adj2 prematurity).tw. (8022)
41 (macular adj2 degeneration).ti. (12629)
42 (laser or photocoagulation or intravitreal or VEGF or bevacizumab or injection or dexamethasone).ti. (256784)
43 (neuropathy or nephropathy or vascular or microvascular).ti. (254854)
44 (animal or primate or rat or rats or mouse or mice or pig).ti. (1601933)
45 or/40-44 (2088854)
46 39 not 45 (3261)
47 limit 46 to yr="2000 -Current" (2775)

For peer review only

Annex 3: Keywords for Google Search

Google Search Engine

Searches "All results" – first 10 pages, representing 1000 results to be screened

Combination of one of

1. Government
2. Health department
3. Ministry of health
4. Provincial health department
5. State health department

AND one of:

1. Afghanistan
2. Albania
3. Algeria
4. Andorra
5. Angola
6. Antigua and Barbuda
7. Argentina
8. Armenia
9. Australia
10. Austria
11. Azerbaijan
12. Bahamas
13. Bahrain
14. Bangladesh
15. Barbados
16. Belarus
17. Belgium
18. Belize
19. Benin
20. Bhutan
21. Bolivia
22. Bosnia and Herzegovina
23. Botswana
24. Brazil
25. Brunei Darussalam
26. Bulgaria
27. Burkina Faso
28. Burundi
29. Cabo Verde
30. Cambodia
31. Cameroon
32. Canada
33. Central African Republic
34. Chad
35. Chile
36. China
37. Colombia
38. Comoros
39. Congo
40. Cook Islands
41. Costa Rica
42. Cote d'Ivoire
43. Croatia
44. Cuba
45. Cyprus
46. Czechia
47. Democratic People's Public of Korea
48. Democratic Republic of Congo
49. Denmark
50. Djibouti
51. Dominica
52. Dominican Republic
53. Ecuador
54. Egypt
55. El Salvador
56. Equatorial Guinea
57. Eritrea
58. Estonia
59. Eswatini
60. Ethiopia
61. Fiji
62. Finland
63. France
64. Gabon
65. Gambia
66. Georgia
67. Germany
68. Ghana
69. Greece
70. Grenada
71. Guatemala
72. Guinea
73. Guinea-Bissau
74. Guyana
75. Haiti
76. Honduras
77. Hungary
78. Iceland
79. India
80. Indonesia
81. Iran
82. Iraq
83. Ireland
84. Israel
85. Italy
86. Jamaica
87. Japan
88. Jordan
89. Kazakhstan
90. Kenya
91. Kiribati
92. Kuwait
93. Kyrgyzstan
94. Lao People's Democratic Republic
95. Latvia
96. Lebanon
97. Lesotho
98. Liberia
99. Libya
100. Lithuania
101. Luxembourg
102. Madagascar
103. Malawi
104. Malaysia
105. Maldives
106. Mali
107. Malta
108. Marshall Islands
109. Mauritania
110. Mauritius
111. Mexico
112. Federated States of Micronesia
113. Monaco
114. Mongolia
115. Montenegro
116. Morocco
117. Mozambique
118. Myanmar
119. Namibia
120. Nauru
121. Nepal
122. Netherlands
123. New Zealand
124. Nicaragua
125. Niger
126. Nigeria
127. Niue
128. North Macedonia
129. Norway
130. Oman
131. Pakistan
132. Palau
133. Panama
134. Papua New Guinea
135. Paraguay
136. Peru
137. Philippines
138. Poland
139. Portugal
140. Qatar
141. Republic of Korea
142. Republic of Moldova
143. Romania
144. Russian Federation

For peer review only

For peer review only - http://bmjopen.bmj.com/site/about/guidelines.xhtml

145. Rwanda
146. Saint Kitts and Nevis
147. Saint Lucia
148. Saint Vincent and the Grenadines
149. Samoa
150. San Marino
151. Sao Tome and Principe
152. Saudi Arabia
153. Senegal
154. Serbia
155. Seychelles
156. Sierra Leone
157. Singapore
158. Slovakia
159. Slovenia
160. Solomon Islands
161. Somalia
162. South Africa
163. South Sudan
164. Spain
165. Sri Lanka
166. Sudan
167. Suriname
168. Sweden
169. Switzerland
170. Syrian Arab Republic
171. Tajikistan
172. Thailand
173. Timor-Leste
174. Togo
175. Tonga
176. Trinidad and Tobago
177. Tunisia
178. Türkiye
179. Turkmenistan
180. Tuvalu
181. Uganda
182. Ukraine
183. United Arab Emirates
184. United Kingdom
185. United Republic of Tanzania
186. United States of America
187. Uruguay
188. Uzbekistan
189. Vanuatu
190. Venezuela
191. Vietnam
192. Yemen
193. Zambia
194. Zimbabwe
195. American Samoa
196. Bermuda
197. French Polynesia
198. Greenland
199. Hong Kong
200. Palestine
201. Puerto Rico
202. Tokelau

For peer review only

For peer review only

For peer review only - http://bmjopen.bmj.com/site/about/guidelines.xhtml

Annex 4: Keywords for individual website searches

1. Diabetic eye screening
2. Diabetic retinopathy screening
3. Diabetic retinopathy screening AND coverage
4. Retina screening coverage
5. Retina screening AND adherence
6. Retina screening AND uptake

For peer review only

Annex 5: Decision tree for inclusion of retina screening coverage for people with diabetes data presented per country for the preferred and alternative data sources for the WHO Definition (shown in Box 1)

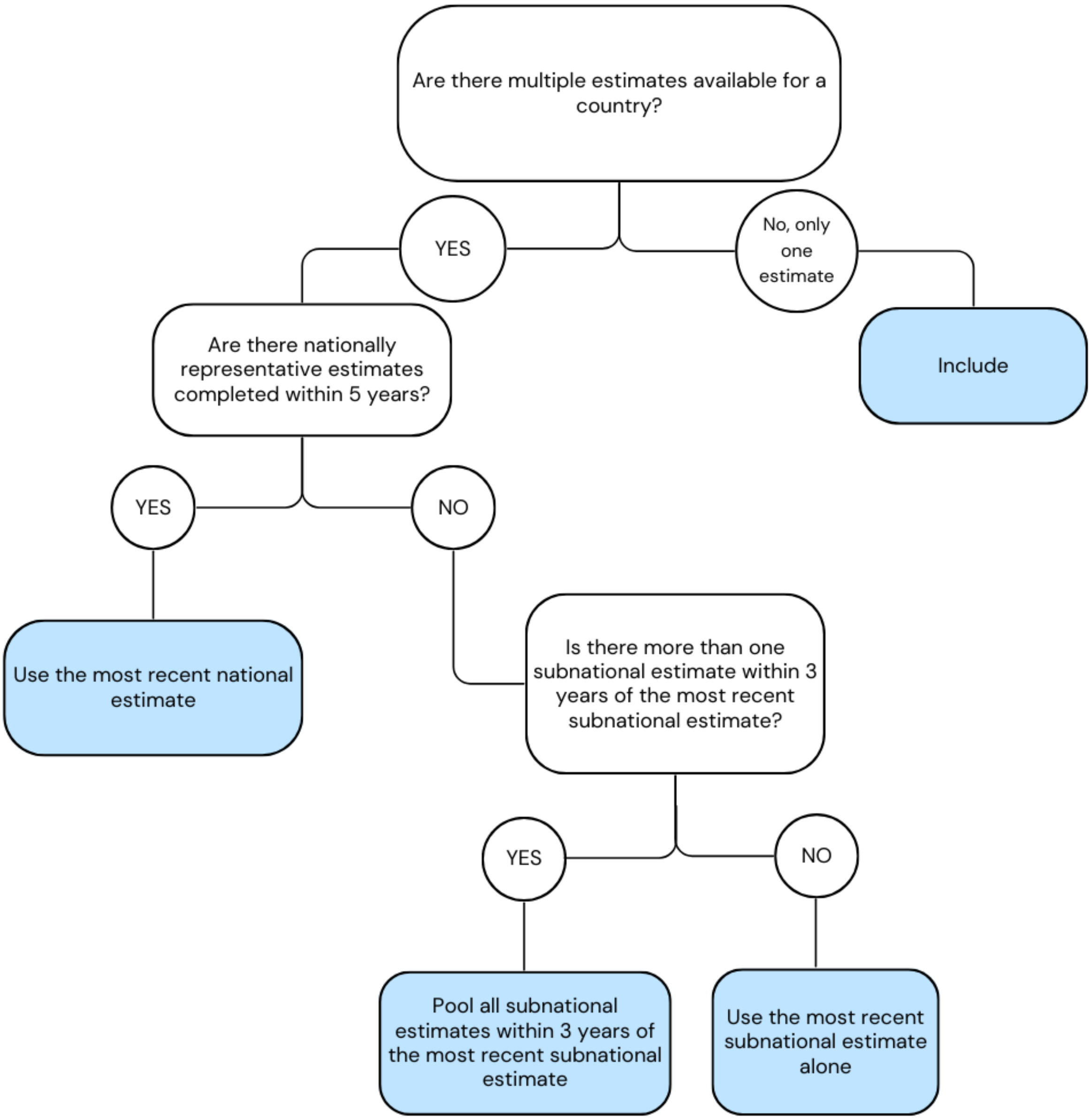

BMJ Open

What is the coverage of retina screening services for people with diabetes? Protocol for a systematic review and meta-analysis

Journal:	*BMJ Open*
Manuscript ID	bmjopen-2023-081123.R1
Article Type:	Protocol
Date Submitted by the Author:	18-Dec-2023
Complete List of Authors:	Chabba, Nimisha; University of Auckland, School of Optometry and Vision Science<br>Silwal, Pushkar; University of Auckland, School of Optometry and Vision Science<br>Bascaran, Covadonga ; London School of Hygiene and Tropical Medicine International Centre for Eye Health,<br>McCormick, Ian; London School of Hygiene and Tropical Medicine, International Centre for Eye Health<br>Goodman, Lucy; University of Auckland, School of Optometry and Vision Science<br>Gordon, Iris; London School of Hygiene & Tropical Medicine, International Centre for Eye Health<br>Burton, Matthew J; London School of Hygiene & Tropical Medicine, International Centre for Eye Health; Moorfields Eye Hospital NHS Foundation Trust and UCL Institute of Ophthalmology, National Institute for Health Research Biomedical Research Centre for Ophthalmology<br>Keel, Stuart ; World Health Organization, Department of Noncommunicable Disease<br>Evans, Jennifer; London School of Hygiene & Tropical Medicine, International Centre for Eye Health<br>Ramke, Jacqueline; University of Auckland, School of Optometry and Vision Science; London School of Hygiene & Tropical Medicine, International Centre for Eye Health
**Primary Subject Heading**:	Diabetes and endocrinology
Secondary Subject Heading:	Global health, Ophthalmology
Keywords:	Diabetic retinopathy < DIABETES & ENDOCRINOLOGY, Mass Screening, General diabetes < DIABETES & ENDOCRINOLOGY

SCHOLARONE™
Manuscripts

What is the coverage of retina screening services for people with diabetes? Protocol for a systematic review and meta-analysis

Nimisha Chabba[1]

Pushkar Silwal[1]

Covadonga Bascaran[2]

Ian McCormick[2]

Lucy Goodman[1]

Iris Gordon[2]

Matthew J Burton[2,3]

Stuart Keel[4]

Jennifer R Evans[2]

Jacqueline Ramke[1,2]

1. School of Optometry and Vision Science, University of Auckland, Auckland, New Zealand
2. International Centre for Eye Health, London School of Hygiene & Tropical Medicine, London, UK
3. National Institute for Health Research Biomedical Research Centre for Ophthalmology at Moorfields Eye Hospital NHS Foundation Trust and UCL Institute of Ophthalmology, London, UK
4. Department of Noncommunicable Disease, World Health Organization, Geneva, Switzerland

Corresponding Author:

Nimisha Chabba

School of Optometry and Vision Science, University of Auckland, Auckland, New Zealand

nimisha.chabba@auckland.ac.nz

For peer review only

Abstract

Introduction

Diabetic retinopathy is a leading cause of vision impairment globally. Vision loss from diabetic retinopathy can generally be prevented by early detection and timely treatment. The World Health Organization (WHO) included a measure of service access for diabetic retinopathy as a core indicator in the Eye Care Indicator Menu launched in 2022: *Retina screening coverage for people with diabetes*. The aim of this review is to provide a comprehensive global and regional summary of the available information on retina screening coverage for people with diabetes.

Methods and analysis

A search will be conducted in five databases without language restrictions for studies from any country reporting retina screening coverage for adults with any type of diabetes at the national or subnational level using data collected since 1 January 2000 until the search date. We will also seek reports and coverage statistics from government websites of all WHO member states. Two investigators will independently screen studies, extract relevant data and assess risk of bias of included studies. The review will be reported using the Preferred Reporting Items for Systematic Review and Meta-Analysis guideline. We will summarise the range of coverage definitions reported across included studies and present the median retina screening coverage in WHO regions and by World Bank country income level. Depending on the availability of data, we will conduct meta-analysis to assess disparities in retina screening coverage for people with diabetes by factors in the PROGRESS framework (Place of residence, Race/ethnicity/culture/language, Occupation, Gender/sex, Religion, Education, Socioeconomic status, and Social capital).

Ethics and dissemination

This review will only include published data thus no ethical approval will be sought. The findings of this review will be published in a peer-reviewed journal and presented at relevant conferences.

Registration

OSF registration 17/10/2023: https://osf.io/k5p69

Strengths and limitations of this study

- A strength of this review is the comprehensive search, designed by an experienced information specialist, and without any language or geographical restrictions, across several databases for reports published from 1 January 2000 until the search date.
- In addition, an extensive and systematic search of government websites (grey literature) will be undertaken and field experts will be requested to nominate additional potentially relevant reports.
- A further strength is that study selection, data extraction and quality assessment will be conducted by two investigators independently.
- A potential limitation is that anticipated heterogeneity in the definitions of the outcome measure, reporting period, and method of data collection may preclude quantitative synthesis of results.

For peer review only - http://bmjopen.bmj.com/site/about/guidelines.xhtml

Introduction

Diabetic retinopathy is a leading cause of vision impairment globally, and the only one of the five leading causes that increased in prevalence in the last 30 years (1). Most of the vision impairment caused by diabetic retinopathy is avoidable through early detection and timely treatment (2, 3). However, vision that has already been lost due to diabetic retinopathy cannot be restored (4). Many countries have retina screening programmes for diabetic retinopathy (also called diabetic eye screening or diabetic retinopathy screening) that are administered at a national-, regional- or facility-level and aim to identify all patients with sight-threatening retinopathy, with subsequent treatment by the same service or via referral to (generally co-located) treatment services (5).

Diabetic retinopathy screening programmes will only be successful in reducing vision loss if most people with diabetes are routinely screened and referred for treatment where indicated. The recommended screening interval varies between countries (e.g., one year in Kenya, 1-2 years in England and 2-3 years in New Zealand) (5, 6). Regardless of the recommended period, available evidence confirms that attendance at retinal screening is suboptimal, with services being less accessible for some population groups (7, 8). People who experience delayed or reduced access to screening will be less likely to be offered timely initiation of treatment should they require it (9).

A measure for diabetic retinopathy service access: *Retina screening coverage for people with diabetes* was recently proposed by the World Health Organization (WHO) as a core indicator for eye health (10). This indicator uses routinely collected health facility data or population-based surveys to measure the percentage of people with known/ diagnosed diabetes who undertake a retina examination at the interval recommended and defined in nationally adopted guidelines. In countries without a recommended screening interval, WHO recommends screening every two years. The way that studies define or report coverage varies. For example, while some studies report coverage at a specific point in time from cross-sectional facility-based data (11) or self-reported service use in survey data (12), others report the proportion of the enrolled diabetic population who attend all expected screening events within a longer period of time (e.g. 5 years of follow-up (13, 14)).

Measuring retina screening coverage is essential to help monitor progress towards the prevention of sight-threatening diabetic retinopathy (15). WHO recently included this indicator in the District Health Information Systems, version 2 (DHIS2) to support the integration and systematic collection of eye care data into facility level reporting systems (16). To date, retina screening coverage for people with diabetes has not been summarised systematically at the global or regional level.

The aim of this review is to provide a comprehensive global and regional summary of the available information on retina screening coverage for people with diabetes.

Objectives

The objectives of this review are to:

(1) Summarise the different ways retina screening coverage for people with diabetes has been defined in published reports.
(2) Summarise retina screening coverage for people with diabetes by World Bank country income level and WHO regions.
(3) Compare disparities in retina screening coverage for people with diabetes by factors in the PROGRESS framework (i.e., between subgroups within any of: Place of residence, Race/ethnicity/culture/language, Occupation, Gender/sex, Religion, Education, Socioeconomic status, and Social capital) (17).

For peer review only - http://bmjopen.bmj.com/site/about/guidelines.xhtml

Methods and analysis

This protocol is reported in accordance with the Preferred Reporting Items for Systematic Reviews and Meta-Analysis Protocols (PRISMA-P) checklist (18, 19). The completed PRISMA-P checklist is presented in supplementary annex 1. The protocol has been registered with the Open Science Framework registry and can be viewed at: https://osf.io/k5p69.

Eligibility criteria

Population and Context

We will include reports from any country reporting retina screening coverage for adults for any type of diabetes. There will be no restrictions on patient characteristics such as sex, ethnicity, duration of diabetes or location. We will exclude studies reporting outcomes only in children (aged 18 years or below) due to the higher likelihood of people <18 years having Type 1 diabetes, with a delayed first screening recommendation. We will exclude studies reporting outcomes only for people with pre-diabetes, and studies reporting outcomes only for people with gestational diabetes because it is mainly a temporary type of diabetes with different screening recommendations.

Type of studies

- Type of report: We will include published (peer reviewed) literature where the full report is published i.e., we will exclude reports with only an abstract available, as well as editorials, protocols and reports of pilot studies. We will also exclude systematic reviews or other evidence syntheses but will examine the reference list of relevant reviews to identify potentially relevant primary studies. We will include grey literature from governments that meet our other inclusion criteria.
- Source of data: We will include data derived from the following sources:
    - population-based research studies;
    - facility-based data collected routinely or *de novo;*
    - service activity or payment data from Ministries of Health or health insurance databases.

  These sources of data may be reported at the national or subnational (state/province/district) level. We will exclude studies reporting data where the sampling is not representative of the national or state/province/district level.
- Study design: We will include cross-sectional studies, cohort studies, randomised controlled trials (for the control arm[s]) and non-randomised intervention studies (for the control arm[s]). We will exclude studies only reporting modelled data.
- Other: Due to the changing nature of the health system over time, we will limit our review to data collected since 1 January 2000. We will exclude studies reporting data exclusively during the peak of the COVID-19 pandemic (2020 and 2021) due to the range of responses and restrictions across the world that reduced health service delivery. If an included study reports estimates from both COVID-19 and non-COVID-19 years, we will include data from the non-COVID-19 period. Our search will have no language restrictions and every effort will be made to translate reports in languages other than English.

Outcome

We will include studies that report a measure of retina screening coverage that includes a numerator and denominator value at a national, or state/province/district level, regardless of the screening interval used (e.g., annual, biennial). We will also include studies that report the number of people with diabetes who attend all of their expected retina screening appointments during a defined follow-up period. The screening could involve a retinal examination by a relevant health practitioner or where an attempt is made to image the retina of a patient by digital photography to determine the presence (and grading) of diabetic retinopathy by a human grader and/or by artificial intelligence. We will exclude studies reporting a coverage proportion without also reporting at least one of either the numerator or denominator and a defined screening interval or time period. We will also exclude studies only reporting attendance at appointments subsequent to retinal screening (e.g., referral to an eye clinic for further assessment).

For peer review only - http://bmjopen.bmj.com/site/about/guidelines.xhtml

When a potentially eligible study only reports coverage disaggregated by population groups and not overall, we will cross-check our included studies to determine if the overall coverage proportion is available in a separate publication; in the event it is not, we will contact study authors to request the overall coverage. If the overall coverage can be provided, we will include the study. In the event the overall coverage cannot be provided, we will include the study only if the population groups for which the data are disaggregated are relevant to the PROGRESS framework, and this study will be considered for analysis of objective 1 and 3 but not objective 2. For example, we will include a study disaggregating coverage for immigrants and non-immigrants, but not a study disaggregating coverage for people with different levels of vision difficulty.

Information sources and search strategy

We will search MEDLINE (Ovid), Cochrane Central Register of Controlled Trials (CENTRAL), Embase, Global Health (Ovid), Emerging Sources Citation Index (ESCI), Science Citation Index Expanded (SCI-EXPANDED), and Social Sciences Citation Index (SSCI) on Web of Science (Clarivate) for reports using data collected since 1 January 2000 using a search strategy developed by an experienced Information Specialist (IG). Our search strategy is included in supplementary annex 2. We will download and de-duplicate the results in EndNote, and then export the results into Covidence (Veritas Health Innovation, Melbourne, Australia. Available at www.covidence.org) for screening. We will examine reference lists of all included reports to identify further potentially relevant reports.

We will search for grey literature in the form of government reports of retina screening coverage at the national or subnational level. Grey literature reports will be searched for by one investigator (NC); another investigator (LG) will repeat the searches for 20 countries to ensure the consistency of relevant results. Government websites, including that of national and provincial government health departments, of the 194 World Health Organization member states and eight associate countries and territories (American Samoa, Bermuda, French Polynesia, Greenland, Hong Kong, Palestine, Puerto Rico, and Tokelau) will be identified and searched for relevant reports and coverage statistics. The process for systematically collecting government reports will be based on the grey literature search framework outlined by Godin et al (20). First, to identify the relevant government websites, a Google search will be conducted using the search strategy in supplementary annex 3. The first ten pages of each search (representing 100 results) will be reviewed, using the title and short text underneath. The relevant websites' name/organisation, country and URL will be entered into an Excel spreadsheet. Then, each of these websites will be searched using the search feature and a combination of keywords included in supplementary annex 4; when the search feature is not available, the website will be scanned for relevant information. When the search feature is available, results will be reviewed using the title and short text underneath and until no more relevant items are identified on a following page. All potentially relevant reports will be exported into Covidence and de-duplicated for title and abstract screening.

Selection

For published literature and grey literature, all title and abstracts (for published literature) or short text (for grey literature) will be screened by two investigators independently using Covidence systematic review management software (Veritas Health Innovation, Melbourne, Australia; available at www.covidence.org). Full-texts of all potentially relevant publications will then be acquired and assessed by two investigators independently to establish eligibility for inclusion into the review. Any disagreements in the screening of search results will be resolved by discussion and consultation with a third investigator as needed.

Following the selection process of published and grey literature, field experts—including clinicians, academics, non-governmental staff, the Diabetic Retinopathy Work Group of the International Agency for the Prevention of Blindness (IAPB) and WHO representatives—will be provided with a list of the included reports and requested to nominate further potentially relevant reports. An Excel spreadsheet will be used to track who has been contacted, who has responded, the need for a follow-up message, and identified reports.

Data collection and data items

Data extraction will be performed in Covidence. The data extraction form will be piloted by two investigators on five studies and modifications undertaken as required. The following details of each report will be extracted by two investigators independently. Any discrepancies will be resolved by discussion, and with a third reviewer if

For peer review only - http://bmjopen.bmj.com/site/about/guidelines.xhtml

For peer review only

necessary. In the event of unclear outcome data, two attempts will be made to contact the corresponding author on email.

- ***Study characteristics***: country/countries of study, type of report, source of data, study design, administrative region of study (when sub-national e.g., province, district, state), year(s) of data collection, number of participants, overall participation rate, extent of missing data.
- ***Participant characteristics***: inclusion criteria (age, diabetes type), age (median/mean and range), diabetes type, diabetic retinopathy status, vision impairment status (21), available information across any PROGRESS dimension (outlined above) (17).
- ***Outcome(s)***: definition of screening coverage (numerator, denominator, screening interval), period of follow-up (if applicable), retina screening coverage result (aggregated and if disaggregated by diabetes type, diabetic retinopathy status or any equity/PROGRESS dimensions (outlined above) (17)).

Outcomes

The primary outcome measure is retina screening coverage for people with diabetes. This is defined in a range of ways. We will include the following broad categories of outcomes and will expand this list based on what is identified during the search.

Box 1: Anticipated definitions of retina screening coverage for people with diabetes

**Definition category** (Reference/example)	**Details**	
WHO definition and data sources (10)	**Data source 1: Preferred data source = facility**	***Definition***: the proportion of people with diabetes who undertook a retina examination at the recommended interval.<br>***Numerator***= Number of people with diabetes who undertook a retina examination at the recommended interval.<br>***Denominator***= Total number of people registered in a facility or, if a register is not available, the estimated prevalence of diabetes in the population covered by a facility.<br>***Interval***= As defined in national screening guidelines or biennially if not outlined in a guideline.
	**Data source 2: Alternative data source = population-based surveys***	***Numerator***= Number of people in the survey who self-report attendance at an eye check for diabetes.<br>***Denominator***= Number of people in the survey with diabetes (either self-reported or confirmed with testing).<br>***Interval***= Either not specified (e.g., the participant is asked when they last attended a check) or a specified period (e.g., the participant is asked whether they attended a check within the last 12 months / 2 years / other time period).
Other definitions	Cohort from facility-based data (14)	***Numerator***= Number of people in the cohort who had attended all of the expected retinal screening appointments (as per the national guideline or biennially) during the follow-up period.<br>***Denominator***= Total number of people registered with diabetes OR if that is not available, the number of people with diabetes who had at least one retinal screening appointment during the follow-up period.<br>***Interval***= As defined in national screening guidelines or biennially if not outlined in a guideline.
	Attendance following diabetes diagnosis (22)	***Numerator***= Number of people with newly diagnosed diabetes who attended the first retinal screening appointment.<br>***Denominator***= Total number of people registered with newly diagnosed diabetes.<br>***Interval***= Within a specified period e.g., within 12 months of diabetes diagnosis.

*The WHO definition requires facility data. For this alternative data source, we have proposed this definition and will reflect on variations of it in reporting of objective 1.

For peer review only - http://bmjopen.bmj.com/site/about/guidelines.xhtml

Risk of bias in individual studies

Two investigators will independently assess the risk of bias in each included study. Any conflict in relation to the appraisal will be discussed between the two investigators and resolved with a third investigator if necessary. Relevant Joanna Briggs Institute (JBI) critical appraisal tools will be used to evaluate each included study (including tools for cohort studies and prevalence studies) (23).

Assessment of heterogeneity

Clinical heterogeneity will be assessed by comparing key participant characteristics at the study level (e.g., age, sex, diabetes type, diabetic retinopathy status). Methodological heterogeneity will also be considered, including variation in the specific definition of the numerator and denominator as well as the reporting period and method of data collection. If meta-analysis is possible, we will assess statistical heterogeneity by inspecting forest plots and the I^2 and τ^2 statistics to examine the proportion of heterogeneity across studies that is due to chance. If high levels of heterogeneity are detected (for example I^2 >50%) and there are sufficient data available (for example, meta-analysis including more than 10 studies), we will explore likely sources of this heterogeneity via meta-regression.

Data synthesis

We will take an inclusive approach for objective 1 and will then determine the potential for synthesis of results reporting the WHO definition (Box 1) for objective 2, before exploring disparities within all included studies in objective 3.

To achieve objective 1, we will outline the full range of definitions used across included studies and summarise the study characteristics of each definition, including geographic (country, national vs sub-national), source of data, study design, inclusion criteria and extent of data missingness.

To achieve objective 2, in countries where estimates on retina screening coverage for people with diabetes using the WHO definition are available from multiple sources, we will establish the country estimate according to the decision tree included in supplementary annex 5. We will first follow the process separately for estimates generated for the preferred and alternative data sources for the WHO definition (facility- and survey-based respectively, see Box 1). The process outlined in the decision tree prioritises more recent studies and studies with nationally representative sampling frames. Where no nationally representative estimates are available, we will pool two or more subnational estimates if the definitions of the outcome are comparable, and the estimates are close enough in value that the pooled result provides a meaningful measure of retina screening coverage for people with diabetes. We will combine these subnational estimates from the same country using an inverse variance weighted average, using the metagen command from the meta package in R. If two sources of data are then available for a country, we will prioritise facility data in countries with organised retina screening programmes and accurate diabetes registries, and survey data in countries with opportunistic screening or with no centralised registries. We will document the extent to which these judgements affect the final country estimates by a sensitivity analysis.

We will use the country estimates to calculate the median, interquartile range (IQR) and range of retina screening coverage for each WHO region and World Bank country income level (24), stratified by screening interval (e.g., 1- 2- and 3-years). We will also compare retina screening coverage estimates over time where comparable sampling frames have been used in repeat studies.

To achieve our third objective, and depending on availability of data, we will calculate risk ratios comparing coverage in different groups, specifically:

- diabetes type (type 2 versus type 1)
- duration of diabetes (per year increase)
- diabetic retinopathy status (present versus absent)

and PROGRESS categories:

For peer review only - http://bmjopen.bmj.com/site/about/guidelines.xhtml

- place of residence (e.g., rural versus urban)
- ethnicity (e.g., non-dominant versus dominant)
- occupation status (e.g., not-employed versus employed).
- gender/sex (e.g., female versus male)
- religion (e.g., non-dominant versus dominant)
- education level (e.g., no educational qualification versus school level qualification versus further educational qualification)
- socioeconomic status (e.g., high versus low area level deprivation)
- social capital (e.g., low versus high social capital)

We will plot these risk ratios in separate forest plots and combine values where appropriate i.e., where we judge that pooling will provide a meaningful measure of comparative risk (see assessment of heterogeneity section above), using the metagen command from the meta package in R. If there are sufficient data available (for example, meta-analysis including 10 or more studies), we will also assess small study effects, one of which may be publication bias, by preparing a funnel plot (25), which is a scatter plot of effect size versus precision (standard error).

Certainty assessment

Currently there is no formal guidance from GRADE regarding the assessment of certainty in systematic reviews and meta-analysis of prevalence studies (26). Therefore, we do not plan to assess certainty formally but we will consider the GRADE domains i.e. risk of bias, inconsistency, imprecision, indirectness and publication bias when drawing conclusions from the available evidence (27).

Patient and public involvement

Patients and the public will not be involved in the conduct of the systematic review and meta-analysis, as we plan to review existing published literature and publicly available reports only.

Ethics and dissemination

This review will only include published data thus no ethical approval will be sought. The findings of this review will be published in a peer-reviewed journal and presented at relevant conferences.

For peer review only

For peer review only - http://bmjopen.bmj.com/site/about/guidelines.xhtml

Authors' contributions

JR conceived the idea for the review. NC and JR drafted and revised the protocol with suggestions from PS, CB, LG, IM, MJB, SK and JE. IG constructed the search.

Funding

NC is supported by a University of Auckland Doctoral Scholarship. PS is supported by a Buchanan Charitable Foundation Postdoctoral Fellowship. MJB is supported by the Wellcome Trust (207472/Z/17/Z).

Competing interests

None declared.

Word Count

2884

ORCID iDs

Nimisha Chabba https://orcid.org/0009-0009-2863-7322

Pushkar Silwal https://orcid.org/0000-0001-6100-090X

Covadonga Bascaran https://orcid.org/0000-0002-5662-3325

Ian McCormick https://orcid.org/0000-0002-7360-3844

Lucy Goodman https://orcid.org/0000-0002-3533-7717

Iris Gordon https://orcid.org/0000-0001-8143-8132

Matthew J Burton https://orcid.org/0000-0003-1872-9169

Stuart Keel https://orcid.org/0000-0001-6756-348X

Jennifer R Evans https://orcid.org/0000-0002-6137-2030

Jacqueline Ramke http://orcid.org/0000-0002-5764-1306

For peer review only

For peer review only - http://bmjopen.bmj.com/site/about/guidelines.xhtml

References

(1) Steinmetz JD, Bourne RRA, Briant PS, Flaxman SR, Taylor HRB, Jonas JB, et al. Causes of blindness and vision impairment in 2020 and trends over 30 years, and prevalence of avoidable blindness in relation to VISION 2020: the Right to Sight: an analysis for the Global Burden of Disease Study. The Lancet Global Health. 2021;9(2):e144–60.

(2) Burton MJ, Ramke J, Marques AP, Bourne RRA, Congdon N, Jones I, et al. The Lancet Global Health Commission on Global Eye Health: vision beyond 2020. The Lancet Global Health. 2021;9(4):e489–551.

(3) Vujosevic S, Aldington SJ, Silva P, Hernández C, Scanlon P, Peto T, et al. Screening for diabetic retinopathy: new perspectives and challenges. The Lancet Diabetes & Endocrinology. 2020;8(4):337–47.

(4) The International Agency for the Prevention of Blindness. Diabetic Retinopathy. 2023. https://www.iapb.org/learn/resources/diabetic-retinopathy/ (accessed 18 June 2023).

(5) Das T, Takkar B, Sivaprasad S, Thanksphon T, Taylor H, Wiedemann P, Nemeth J, Nayar PD, Rani PK, Khandekar R. Recently updated global diabetic retinopathy screening guidelines: commonalities, differences, and future possibilities. Eye. 2021;35(10):2685-98.

(6) The College of Optometrists. Changes to the NHS Diabetic Eye Screening Programme in England. The College of Optometrists. https://www.college-optometrists.org/news/2023/october/changes-nhs-diabetic-eye-screening-programme (accessed 14 October 2023)

(7) Wong TY, Sabanayagam C. Strategies to tackle the global burden of diabetic retinopathy: from epidemiology to artificial intelligence. Ophthalmologica. 2020;243(1):9-20.

(8) Silwal PR, Lee AC, Squirrell D, Zhao J, Harwood M, Vincent AL, Murphy R, Ameratunga S, Ramke J. Use of public sector diabetes eye services in New Zealand 2006–2019: Analysis of national routinely collected datasets. Plos one. 2023;18(5):e0285904.

(9) Ting DS, Cheung GC, Wong TY. Diabetic retinopathy: global prevalence, major risk factors, screening practices and public health challenges: a review. Clinical & experimental ophthalmology. 2016;44(4):260-77.

(10) World Health Organization. Eye care indicator menu (ECIM): a tool for monitoring strategies and actions for eye care provision. Geneva: World Health Organization; 2022.

(11) Wilson A, Baker R, Thompson J, Grimshaw G. Coverage in screening for diabetic retinopathy according to screening provision: results from a national survey in England and Wales. Diabetic medicine. 2004;21(3):271-8.

(12) Foreman J, Keel S, Xie J, Van Wijngaarden P, Taylor HR, Dirani M. Adherence to diabetic eye examination guidelines in Australia: the National Eye Health Survey. Medical Journal of Australia. 2017;206(9):402-6.

(13) Gibson AA, Humphries J, Gillies M, Nassar N, Colagiuri S. Adherence to eye examination guidelines among individuals with diabetes: an analysis of linked health data. Clinical & Experimental Ophthalmology. 2020;48(9):1229-38.

(14) An J, Niu F, Turpcu A, Rajput Y, Cheetham TC. Adherence to the American Diabetes Association retinal screening guidelines for population with diabetes in the United States. Ophthalmic epidemiology. 2018;25(3):257-65.

(15) World Health Organisation Regional Office for Europe. Diabetic retinopathy screening: a short guide. Increase effectiveness, maximise benefits and minimise harm. Copenhagen: WHO Regional Office for Europe, 2020.

(16) World Health Organization. Guidance on the analysis and use of routine health information systems: eye and ear care module. Geneva: World Health Organization; 2023.

(17) O'Neill J, Tabish H, Welch V, Petticrew M, Pottie K, Clarke M, Evans T, Pardo JP, Waters E, White H, Tugwell P. Applying an equity lens to interventions: using PROGRESS ensures consideration of socially stratifying factors to illuminate inequities in health. Journal of clinical epidemiology. 2014;67(1):56-64.

(18) Moher D, Shamseer L, Clarke M, Ghersi D, Liberati A, Petticrew M, Shekelle P, Stewart LA. Preferred Reporting Items for Systematic Review and Meta-Analysis Protocols (PRISMA-P) 2015 statement. Syst Rev. 2015;4(1):1.

(19) Shamseer L, Moher D, Clarke M, Ghersi D, Liberati A, Petticrew M, Shekelle P, Stewart LA, the PRISMA-P Group. Preferred Reporting Items for Systematic Review and Meta-Analysis Protocols (PRISMA-P) 2015: elaboration and explanation. BMJ 2015.349:g7647.

(20) Godin K, Stapleton J, Kirkpatrick SI, Hanning RM, Leatherdale ST. Applying systematic review search methods to the grey literature: a case study examining guidelines for school-based breakfast programs in Canada. Systematic reviews. 2015;4(1):1-0.

(21) World Health Organization. International Classification of Diseases. 11th rev., ICD-11. Geneva: World Health Organization, 2018.

(22) Scanlon PH, Stratton IM, Leese GP, Bachmann MO, Land M, Jones C, Ferguson B, Four Nations Diabetic Retinopathy Screening Study Group. Screening attendance, age group and diabetic retinopathy level at first screen. Diabetic Medicine. 2016;33(7):904-11.

(23) JBI. Critical Appraisal Tools. JBI. 2023. https://jbi.global/critical-appraisal-tools (accessed 20 July 2023).

(24) The World Bank. The world by income and region. 2023. https://datatopics.worldbank.org/world-development-indicators/the-world-by-income-and-region.html (accessed 15 August 2023).

(25) Egger M, Smith GD, Schneider M, Minder C. Bias in meta-analysis detected by a simple, graphical test. BMJ. 1997;315(7109):629-34.

For peer review only - http://bmjopen.bmj.com/site/about/guidelines.xhtml

(26) Borges Migliavaca C, Stein C, Colpani V, Barker TH, Munn Z, Falavigna M, Prevalence Estimates Reviews–Systematic Review Methodology Group (PERSyst). How are systematic reviews of prevalence conducted? A methodological study. BMC medical research methodology. 2020;20:1-9.

(27) Schünemann H. Brożek J. Guyatt G. Oxman A. editors. GRADE handbook for grading quality of evidence and strength of recommendations. Updated October 2013. The GRADE Working Group, 2013. Available from guidelinedevelopment.org/handbook.

For peer review only

For peer review only - http://bmjopen.bmj.com/site/about/guidelines.xhtml

Annex 1: Reporting of PRISMA-P 2015 Items

Section and topic	Item No	Checklist item	Location where reported
**ADMINISTRATIVE INFORMATION**			
Title:			
Identification	1a	Identify the report as a protocol of a systematic review	1
Update	1b	If the protocol is for an update of a previous systematic review, identify as such	N/A
Registration	2	If registered, provide the name of the registry (such as PROSPERO) and registration number	2
Authors:			
Contact	3a	Provide name, institutional affiliation, e-mail address of all protocol authors; provide physical mailing address of corresponding author	1
Contributions	3b	Describe contributions of protocol authors and identify the guarantor of the review	11
Amendments	4	If the protocol represents an amendment of a previously completed or published protocol, identify as such and list changes; otherwise, state plan for documenting important protocol amendments	N/A
Support:			
Sources	5a	Indicate sources of financial or other support for the review	11
Sponsor	5b	Provide name for the review funder and/or sponsor	11
Role of sponsor or funder	5c	Describe roles of funder(s), sponsor(s), and/or institution(s), if any, in developing the protocol	N/A
**INTRODUCTION**			
Rationale	6	Describe the rationale for the review in the context of what is already known	3
Objectives	7	Provide an explicit statement of the question(s) the review will address with reference to participants, interventions, comparators, and outcomes (PICO)	3
**METHODS**			
Eligibility criteria	8	Specify the study characteristics (such as PICO, study design, setting, time frame) and report characteristics (such as years considered, language, publication status) to be used as criteria for eligibility for the review	4
Information sources	9	Describe all intended information sources (such as electronic databases, contact with study authors, trial registers or other grey literature sources) with planned dates of coverage	5
Search strategy	10	Present draft of search strategy to be used for at least one electronic database, including planned limits, such that it could be repeated	Annex 2-4
Study records:			
Data management	11a	Describe the mechanism(s) that will be used to manage records and data throughout the review	5
Selection process	11b	State the process that will be used for selecting studies (such as two independent reviewers) through each phase of the review (that is, screening, eligibility and inclusion in meta-analysis)	5
Data collection process	11c	Describe planned method of extracting data from reports (such as piloting forms, done independently, in duplicate), any processes for obtaining and confirming data from investigators	5-6
Data items	12	List and define all variables for which data will be sought (such as PICO items, funding sources), any pre-planned data assumptions and simplifications	5-6
Outcomes and prioritization	13	List and define all outcomes for which data will be sought, including prioritization of main and additional outcomes, with rationale	5-6
Risk of bias in individual studies	14	Describe anticipated methods for assessing risk of bias of individual studies, including whether this will be done at the outcome or study level, or both; state how this information will be used in data synthesis	7
Data synthesis	15a	Describe criteria under which study data will be quantitatively synthesised	7-8
	15b	If data are appropriate for quantitative synthesis, describe planned summary measures, methods of handling data and methods of combining data from studies, including any planned exploration of consistency (such as I^2, Kendall's τ)	7-8
	15c	Describe any proposed additional analyses (such as sensitivity or subgroup analyses, meta-regression)	7
	15d	If quantitative synthesis is not appropriate, describe the type of summary planned	7
Meta-bias(es)	16	Specify any planned assessment of meta-bias(es) (such as publication bias across studies, selective reporting within studies)	7-8
Confidence in cumulative evidence	17	Describe how the strength of the body of evidence will be assessed (such as GRADE)	8

For peer review only - http://bmjopen.bmj.com/site/about/guidelines.xhtml

Annex 2: Search Strategy

Database: MEDLINE

1. exp Diabetes Mellitus/
2. exp Diabetes Complications/
3. Diabetic Retinopathy/
4. ((diabet$ or proliferative or non-proliferative) adj4 retinopath$).tw.
5. diabetic retinopathy.kw.
6. (diabet$ adj3 (eye$ or vision or visual$ or sight$)).tw.
7. (retinopath$ adj3 (eye$ or vision or visual$ or sight$)).tw.
8. (DR adj3 (eye$ or vision or visual$ or sight$)).tw.
9. (diabet$ adj3 macula$ adj3 oedema).tw.
10. (diabet$ adj3 macula$ adj3 edema).tw.
11. (DMO or DME).tw.
12. or/1-11
13. Vision Screening/
14. 12 and 13
15. Mass Screening/
16. or/3-11
17. 15 and 16
18. 14 or 17
19. ((eye$ or retina or retinal) adj3 (screen$ or exam$ or check$)).tw.
20. (diabet$ adj2 eye adj2 care).tw.
21. (diabet$ adj2 eye adj2 service$).tw.
22. or/19-21
23. 12 and 22
24. 18 or 23
25. (pregnan$ or gestational).ti.
26. artificial intelligence/
27. deep learning/
28. exp machine learning/
29. "neural networks (computer)"/
30. fuzzy logic/
31. (artificial adj1 intelligence).ti.
32. ((deep or machine or transfer) adj2 learning).ti.
33. ((deep or convolutional or neural) adj3 network$).ti.
34. (retinopath$ adj2 prematurity).tw.
35. (macular adj2 degeneration).ti.
36. (laser or photocoagulation or intravitreal or VEGF or bevacizumab or injection or dexamethasone or triamcinolone).ti.
37. (neuropathy or nephropathy or vascular or microvascular).ti.
38. (animal or primate or rat or rats or mouse or mice or pig).ti.
39. or/25-38
40. 24 not 39
41. case reports/
42. (case adj3 report$).tw.
43. or/41-42
44. 40 not 43
45. limit 44 to yr="2000 -Current"

For peer review only

Database: Embase

1. exp diabetes mellitus/
2. diabetic retinopathy/
3. ((diabet$ or proliferative or non-proliferative) adj4 retinopath$).tw.
4. diabetic retinopathy.kw.
5. (diabet$ adj3 (eye$ or vision or visual$ or sight$)).tw.
6. (retinopath$ adj3 (eye$ or vision or visual$ or sight$)).tw.
7. (DR adj3 (eye$ or vision or visual$ or sight$)).tw.
8. (diabet$ adj3 macula$ adj3 oedema).tw.
9. (diabet$ adj3 macula$ adj3 edema).tw.
10. (DMO or DME).tw.
11. or/1-10
12. exp vision test/
13. 11 and 12
14. mass screening/
15. or/2-10
16. 14 and 15
17. 13 or 16
18. ((eye$ or retina or retinal) adj3 (screen$ or exam$ or check$)).tw.
19. (diabet$ adj2 eye adj2 care).tw.
20. (diabet$ adj2 eye adj2 service$).tw.
21. or/18-20
22. 11 and 21
23. 17 or 22
24. (pregnan$ or gestational).ti.
25. artificial intelligence/
26. deep learning/
27. exp machine learning/
28. "neural networks (computer)"/
29. fuzzy logic/
30. (artificial adj1 intelligence).ti.
31. ((deep or machine or transfer) adj2 learning).ti.
32. ((deep or convolutional or neural) adj3 network$).ti.
33. (retinopath$ adj2 prematurity).tw.
34. (macular adj2 degeneration).ti.
35. (laser or photocoagulation or intravitreal or VEGF or bevacizumab or injection or dexamethasone).ti.
36. (neuropathy or nephropathy or vascular or microvascular).ti.
37. (animal or primate or rat or rats or mouse or mice or pig).ti.
38. or/24-37
39. 23 not 38
40. exp case report/
41. (case adj3 report$).tw.
42. or/40-41
43. 39 not 42
44. limit 43 to conference abstract status
45. 43 not 44
46. limit 45 to yr="2000 -Current"

For peer review only

For peer review only - http://bmjopen.bmj.com/site/about/guidelines.xhtml

Database: Global Health

1. Screening/
2. Diabetic Retinopathy/
3. ((diabet$ or proliferative or non-proliferative) adj4 retinopath$).tw.
4. (diabet$ adj3 (eye$ or vision or visual$ or sight$)).tw.
5. (retinopath$ adj3 (eye$ or vision or visual$ or sight$)).tw.
6. (DR adj3 (eye$ or vision or visual$ or sight$)).tw.
7. (diabet$ adj3 macula$ adj3 oedema).tw.
8. (diabet$ adj3 macula$ adj3 edema).tw.
9. (DMO or DME).tw.
10. or/2-9
11. 1 and 10
12. Diabetes Mellitus/
13. ((eye$ or retina or retinal) adj3 (screen$ or exam$ or check$)).tw.
14. (diabet$ adj2 eye adj2 care).tw.
15. (diabet$ adj2 eye adj2 service$).tw.
16. or/13-15
17. 10 or 12
18. 16 and 17
19. 11 or 18
20. (pregnan$ or gestational).ti.
21. artificial intelligence/
22. exp machine learning/
23. neural networks/
24. fuzzy logic/
25. (artificial adj1 intelligence).ti.
26. ((deep or machine or transfer) adj2 learning).ti.
27. ((deep or convolutional or neural) adj3 network$).ti.
28. (retinopath$ adj2 prematurity).tw.
29. (macular adj2 degeneration).ti.
30. (laser or photocoagulation or intravitreal or VEGF or bevacizumab or injection or dexamethasone).ti.
31. (neuropathy or nephropathy or vascular or microvascular).ti.
32. (animal or primate or rat or rats or mouse or mice or pig).ti.
33. or/20-32
34. 19 not 33
35. case reports/
36. (case adj3 report$).tw.
37. or/35-36
38. 34 not 37
39. limit 38 to yr="2000 -Current"

For peer review only

Database: CENTRAL on Cochrane Library

1. #1 MeSH descriptor: [Diabetes Mellitus] explode all trees
2. #2 MeSH descriptor: [Diabetes Complications] explode all trees
3. #3 MeSH descriptor: [Diabetic Retinopathy] this term only
4. #4 (diabet* or proliferative or non-proliferative) NEAR/4 retinopath*
5. #5 diabet* NEAR/3 (eye* or vision or visual* or sight*)
6. #6 retinopath* NEAR/3 (eye* or vision or visual* or sight*)
7. #7 DR NEAR/3 (eye* or vision or visual* or sight*)
8. #8 (diabet* NEAR/3 macula* NEAR/3 oedema)
9. #9 (diabet* NEAR/3 macula* NEAR/3 edema)
10. #10 DMO or DME
11. #11 #1 or #2 or #3 or #4 or #5 or #6 or #7 or #8 or #9 or #10
12. #12 MeSH descriptor: [Vision Screening] this term only
13. #13 #11 and #12
14. #14 MeSH descriptor: [Mass Screening] this term only
15. #15 #3 or #4 or #5 or #6 or #7 or #8 or #9 or #10
16. #16 #14 and #15
17. #17 #13 or #16
18. #18 (eye* or retina or retinal) NEAR/3 (screen* or exam* or check*)
19. #19 (diabet* NEAR/2 eye NEAR/2 care)
20. #20 (diabet* NEAR/2 eye NEAR/2 service*)
21. #21 #18 or #19 or #20
22. #22 #17 and #21 with Publication Year from 2000 to 2023, in Trials

For peer review only

For peer review only - http://bmjopen.bmj.com/site/about/guidelines.xhtml

Database: Web of Science - Science Citation Index Expanded (SCI-EXPANDED), Social Sciences Citation Index (SSCI), Emerging Sources Citation Index (ESCI)

1. 1: Screen* (Topic)
2. 2: TS=((diabet* or proliferative or non-proliferative) NEAR/4 retinopath*)
3. 3: TS= (diabet* NEAR/3 (eye* or vision or visual* or sight*))
4. 4: TS= (retinopath* NEAR/3 (eye* or vision or visual* or sight*))
5. 5: TS= (DR NEAR/3 (eye* or vision or visual* or sight*))
6. 6: TS= (diabet* NEAR/3 macula* NEAR/3 oedema)
7. 7: TS= (diabet* NEAR/3 macula* NEAR/3 edema)
8. 8: TS= (DMO or DME)
9. 9: #8 OR #7 OR #6 OR #5 OR #4 OR #3 OR #2
10. 10: #9 AND #1
11. 11: TS= Diabetes Mellitus
12. 12: TS= ((eye* or retina or retinal) NEAR/3 (screen* or exam* or check*))
13. 13: TS= (diabet* NEAR/2 eye NEAR/2 care)
14. 14: TS= (diabet* NEAR/2 eye NEAR/2 service*)
15. 15: #12 OR #13 OR #14
16. 16: #11 OR #9
17. 17: #15 AND #16
18. 18: #10 OR #17
19. 19: TI= (pregnan* or gestational)
20. 20: TI= ((deep or machine or transfer) NEAR/2 learning)
21. 21: TI= ((deep or convolutional or neural) NEAR/3 network*)
22. 22: TS= (retinopath* NEAR/2 prematurity)
23. 23: TS= (macular NEAR/2 degeneration)
24. 24: TI= (laser or photocoagulation or intravitreal or VEGF or bevacizumab or injection or dexamethasone or neuropathy or nephropathy or vascular or microvascular or neuropathy or nephropathy or vascular or microvascular)
25. 25: TI=(animal or primate or rat or rats or mouse or mice or pig)
26. 26: #25 OR #24 OR #23 OR #22 OR #21 OR #20 OR #19
27. 27: #18 NOT #26
28. 28: #18 NOT #26 and 2024 or 2023 or 2022 or 2021 or 2020 or 2019 or 2018 or 2017 or 2016 or 2015 or 2014 or 2008 or 2007 or 2006 or 2005 or 2004 or 2003 or 2002 or 2001 or 2000 (Publication Years)

For peer review only

Annex 3: Keywords for Google Search

Google Search Engine

Searches "All results" – first 10 pages, representing 1000 results to be screened

Combination of one of

1. Government
2. Health department
3. Ministry of health
4. Provincial health department
5. State health department

AND one of:

1. Afghanistan
2. Albania
3. Algeria
4. Andorra
5. Angola
6. Antigua and Barbuda
7. Argentina
8. Armenia
9. Australia
10. Austria
11. Azerbaijan
12. Bahamas
13. Bahrain
14. Bangladesh
15. Barbados
16. Belarus
17. Belgium
18. Belize
19. Benin
20. Bhutan
21. Bolivia
22. Bosnia and Herzegovina
23. Botswana
24. Brazil
25. Brunei Darussalam
26. Bulgaria
27. Burkina Faso
28. Burundi
29. Cabo Verde
30. Cambodia
31. Cameroon
32. Canada
33. Central African Republic
34. Chad
35. Chile
36. China
37. Colombia
38. Comoros
39. Congo
40. Cook Islands
41. Costa Rica
42. Cote d'Ivoire
43. Croatia
44. Cuba
45. Cyprus
46. Czechia
47. Democratic People's Public of Korea
48. Democratic Republic of Congo
49. Denmark
50. Djibouti
51. Dominica
52. Dominican Republic
53. Ecuador
54. Egypt
55. El Salvador
56. Equatorial Guinea
57. Eritrea
58. Estonia
59. Eswatini
60. Ethiopia
61. Fiji
62. Finland
63. France
64. Gabon
65. Gambia
66. Georgia
67. Germany
68. Ghana
69. Greece
70. Grenada
71. Guatemala
72. Guinea
73. Guinea-Bissau
74. Guyana
75. Haiti
76. Honduras
77. Hungary
78. Iceland
79. India
80. Indonesia
81. Iran
82. Iraq
83. Ireland
84. Israel
85. Italy
86. Jamaica
87. Japan
88. Jordan
89. Kazakhstan
90. Kenya
91. Kiribati
92. Kuwait
93. Kyrgyzstan
94. Lao People's Democratic Republic
95. Latvia
96. Lebanon
97. Lesotho
98. Liberia
99. Libya
100. Lithuania
101. Luxembourg
102. Madagascar
103. Malawi
104. Malaysia
105. Maldives
106. Mali
107. Malta
108. Marshall Islands
109. Mauritania
110. Mauritius
111. Mexico
112. Federated States of Micronesia
113. Monaco
114. Mongolia
115. Montenegro
116. Morocco
117. Mozambique
118. Myanmar
119. Namibia
120. Nauru
121. Nepal
122. Netherlands
123. New Zealand
124. Nicaragua
125. Niger
126. Nigeria
127. Niue
128. North Macedonia
129. Norway
130. Oman
131. Pakistan
132. Palau
133. Panama
134. Papua New Guinea
135. Paraguay
136. Peru
137. Philippines
138. Poland
139. Portugal
140. Qatar
141. Republic of Korea
142. Republic of Moldova
143. Romania
144. Russian Federation

For peer review only

145. Rwanda
146. Saint Kitts and Nevis
147. Saint Lucia
148. Saint Vincent and the Grenadines
149. Samoa
150. San Marino
151. Sao Tome and Principe
152. Saudi Arabia
153. Senegal
154. Serbia
155. Seychelles
156. Sierra Leone
157. Singapore
158. Slovakia
159. Slovenia
160. Solomon Islands
161. Somalia
162. South Africa
163. South Sudan
164. Spain
165. Sri Lanka
166. Sudan
167. Suriname
168. Sweden
169. Switzerland
170. Syrian Arab Republic
171. Tajikistan
172. Thailand
173. Timor-Leste
174. Togo
175. Tonga
176. Trinidad and Tobago
177. Tunisia
178. Türkiye
179. Turkmenistan
180. Tuvalu
181. Uganda
182. Ukraine
183. United Arab Emirates
184. United Kingdom
185. United Republic of Tanzania
186. United States of America
187. Uruguay
188. Uzbekistan
189. Vanuatu
190. Venezuela
191. Vietnam
192. Yemen
193. Zambia
194. Zimbabwe
195. American Samoa
196. Bermuda
197. French Polynesia
198. Greenland
199. Hong Kong
200. Palestine
201. Puerto Rico
202. Tokelau

For peer review only

For peer review only

For peer review only - http://bmjopen.bmj.com/site/about/guidelines.xhtml

Annex 4: Keywords for individual website searches

1. Diabetic eye screening
2. Diabetic retinopathy screening
3. Diabetic retinopathy screening AND coverage
4. Retina screening coverage
5. Retina screening AND adherence
6. Retina screening AND uptake

For peer review only

For peer review only - http://bmjopen.bmj.com/site/about/guidelines.xhtml

Annex 5: Decision tree for inclusion of retina screening coverage for people with diabetes data presented per country for the preferred and alternative data sources for the WHO Definition (shown in Box 1)

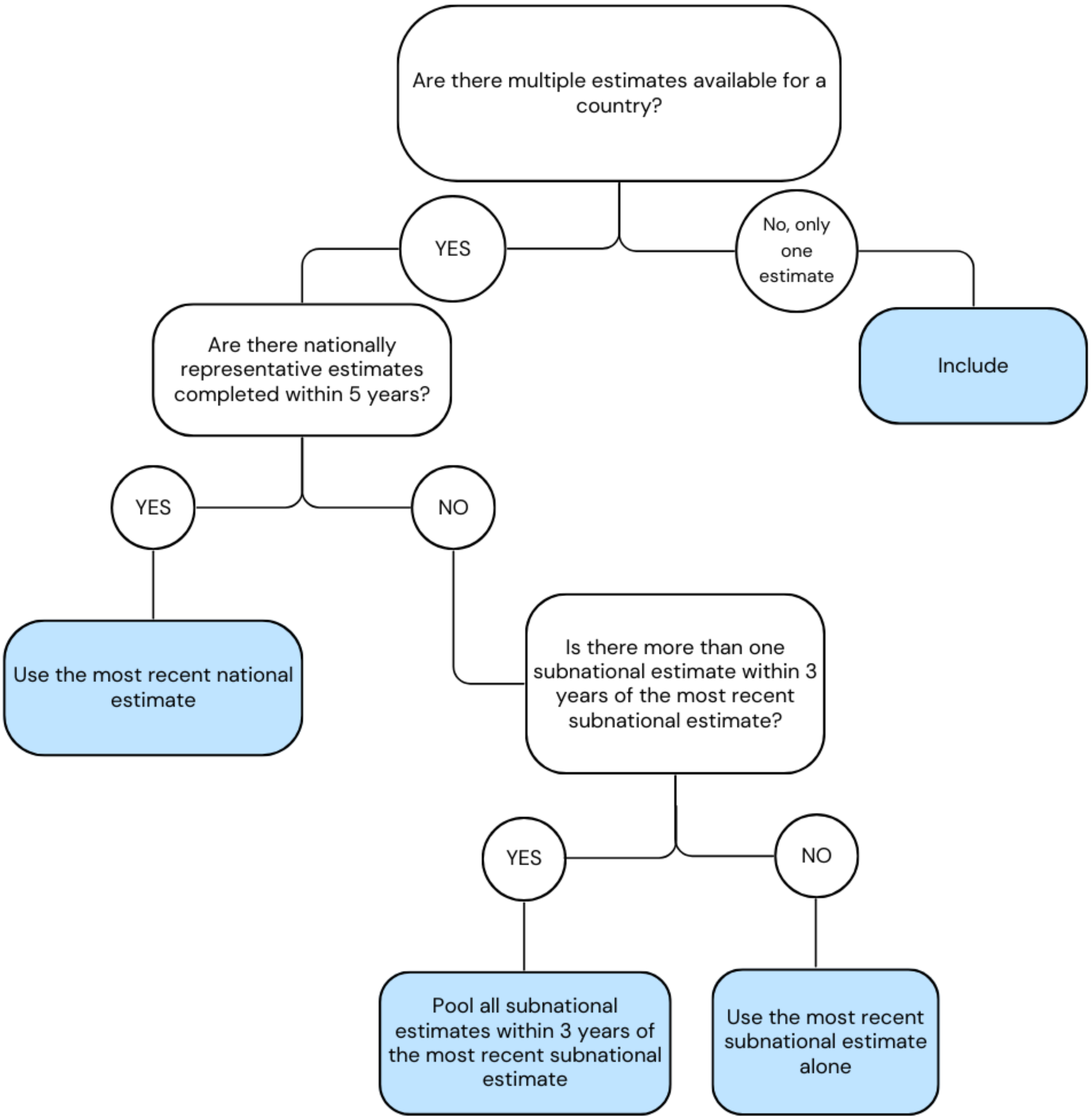

Supplement: Author's manuscript [file bmjopen-2023-081123.draft_revisions.pdf]
